# Can Metabolite- and Transcript-Based Selection for Drought Tolerance in *Solanum tuberosum* Replace Selection on Yield in Arid Environments?

**DOI:** 10.3389/fpls.2020.01071

**Published:** 2020-07-21

**Authors:** Manuela Haas, Heike Sprenger, Ellen Zuther, Rolf Peters, Sylvia Seddig, Dirk Walther, Joachim Kopka, Dirk K. Hincha, Karin I. Köhl

**Affiliations:** ^1^ Max Planck Institute of Molecular Plant Physiology, Potsdam-Golm, Germany; ^2^ Versuchsstation Dethlingen, Landwirtschaftskammer Niedersachsen, Munster, Germany; ^3^ Federal Research Centre for Cultivated Plants, Institute for Resistance Research and Stress Tolerance, Julius-Kühn Institut, Sanitz, Germany

**Keywords:** drought tolerance, least absolute shrinkage and selection operator models, marker-assisted selection, metabolite profiling, multi-environment trials, phenotyping, potato, transcript profiling

## Abstract

Climate models predict an increased likelihood of drought, demanding efficient selection for drought tolerance to maintain yield stability. Classic tolerance breeding relies on selection for yield in arid environments, which depends on yield trials and takes decades. Breeding could be accelerated by marker-assisted selection (MAS). As an alternative to genomic markers, transcript and metabolite markers have been suggested for important crops but also for orphan corps. For potato, we suggested a random-forest-based model that predicts tolerance from leaf metabolite and transcript levels with a precision of more than 90% independent of the agro-environment. To find out how the model based selection compares to yield-based selection in arid environments, we applied this approach to a population of 200 tetraploid *Solanum tuberosum* ssp. *tuberosum* lines segregating for drought tolerance. Twenty-four lines were selected into a phenotypic subpopulation (PP_t_) for superior tolerance based on relative tuber starch yield data from three drought stress trials. Two subpopulations with superior (MP_t_) and inferior (MP_s_) tolerance were selected based on drought tolerance predictions based on leaf metabolite and transcript levels from two sites. The 60 selected lines were phenotyped for yield and drought tolerance in 10 multi-environment drought stress trials representing typical Central European drought scenarios. Neither selection affected development or yield potential. Lines with superior drought tolerance and high yields under stress were over-represented in both populations selected for superior tolerance, with a higher number in PP_t_ compared to MP_t_. However, selection based on leaf metabolites may still be an alternative to yield-based selection in arid environments as it works on leaves sampled in breeder’s fields independent of drought trials. As the selection against low tolerance was ineffective, the method is best used in combination with tools that select against sensitive genotypes. Thus, metabolic and transcript marker-based selection for drought tolerance is a viable alternative to the selection on yield in arid environments.

## Introduction

In the next 30 years, agricultural production must double to ensure global food supply (Stamp and Visser, 2012). Agriculture is predominantly limited by abiotic stresses, in particular drought and unfavorable temperatures, problems that are likely to be aggravated by global climate change (Harrison et al., 2014). In spite of water saving techniques, insufficient water supply will become more frequent because of altered precipitation patterns and an increase of competing water demand by industry and domestic consumption (Monneveux et al., 2012; Monneveux et al., 2013). Combining increased agricultural productivity with sustainable water management thus requires improved selection and phenotyping of drought tolerant or resilient genotypes (Pimentel et al., 1998; Blum, 2013). Drought tolerance in crops is the ability to produce yield with limited water supply (Blum et al., 1989). In wheat, substantial increase in yield under arid conditions has been achieved by selecting for high grain yield potentials (Richards et al., 2014), a concept favored by breeders. However, this approach may have slowed down progress in the breeding of resilient cultivars (Thiry et al., 2016).

Potato (*Solanum tuberosum* L.) is the world’s fourth most important food crop and yields more food calories per unit water than cereals (Monneveux et al., 2013; Slater et al., 2016). However, potato drought tolerance is low because of a shallow root system and a low recuperation capacity after drought (van Loon, 1981; Vos and Groenwold, 1986; Anithakumari et al., 2012; Obidiegwu et al., 2015). Lack of water is the most important yield-limiting stress and yield losses due to drought are predicted to increase by 18–32% in the 21^st^ century (Jones et al., 2003). Conventional breeding strategies in potato depend on rather inefficient phenotypic recurrent selection (Slater et al., 2014a; Gopal, 2015). The introgression of drought tolerance genes from wild relatives of potato and South American land races involves a substantial linkage drag introducing undesirable features (Schafleitner et al., 2007; Cabello et al., 2012). However, European *Solanum tuberosum* ssp. tuberosum cultivars vary significantly for drought tolerance thus providing a genetic basis for drought tolerance breeding (Jefferies and Mackerron, 1987; Wishart et al., 2013; Sprenger et al., 2015; Soltys-Kalina et al., 2016; Aliche et al., 2018). Selection for yield stability is challenging as drought tolerance is a highly polygenic trait and heritability of yield decreases under stress (Bolaños et al., 1993; Slater et al., 2014a). Furthermore, a substantial interaction between tolerance traits and environment renders traits favorable in one environment neutral or even negative in another environment (Tardieu, 2012; Parent and Tardieu, 2014). Drought tolerance breeding thus requires laborious testing of a large number of lines in multi-environment trials. The use of marker-assisted selection (MAS), especially in the early breeding cycles could accelerate progress by decreasing the duration of a breeding cycle from ten to four years (Slater et al., 2016). However, efficient genomic MAS for quantitative characteristics requires large effect QTL or a group of markers linked to alleles with smaller effects (Slater et al., 2014a). Presently, most MAS examples successfully introduced into practical breeding are linked to disease resistance (Slater et al., 2014a). Modern methods of genomic selection require detailed genotyping of the germplasm. This information is missing for many orphan crops. As an alternative to genomic selection, metabolite-marker-based selection has been suggested for well-studied crops like maize and rice, but also for understudied crops like *Ipomoea batatas* (Obata and Fernie, 2012; Riedelsheimer et al., 2012; Degenkolbe et al., 2013; Melandri et al., 2020; Price et al., 2020). In a previous publication, we presented a random-forest model that predicted drought tolerance within a panel of German potato cultivars with an accuracy above 90% (Sprenger et al., 2016; Sprenger et al., 2018). The high accuracy and the fact that prediction was independent of the agro-environment, in which the leaves were sampled, was unexpected. Now, we wanted to know whether this approach would efficiently select tolerant genotypes from an independent population and identify genotypes with increased drought tolerance under a range of typical central European drought scenarios. In previous studies on maize, metabolite-based prediction models and SNP-based prediction models showed a similar range of accuracy for the prediction of biomass-related traits (Riedelsheimer et al., 2012). In the present study, we compared metabolite-based and transcript-based selection (Sprenger et al., 2016; Sprenger et al., 2018) to phenotypic selection based on yield data from a limited set of drought stress trials to mimic selection in an arid environment. For this purpose, we generated a population of lines segregating for drought tolerance and phenotyped it for leaf metabolite and transcript levels and for tuber starch yield in drought stress trials. Based on these data, a phenotypic subpopulation was selected for superior tolerance based on yield data from three trials. Two additional populations with lines of superior or inferior tolerance were selected based on tolerance prediction with a least absolute shrinkage and selection operator (LASSO) model (Friedman et al., 2010). To find out, whether the quality of the prediction based on metabolite/transcript markers is similar to the prediction based on starch yield data from a limited set of trials, we characterized the drought tolerance of the selected lines in 10 multi-environment trials representing typical central European drought scenario. An overview of the workflow can be found in **Data Sheet 1**.

## Material and Methods

### Creation of a Segregating Population

**Figure 1:**
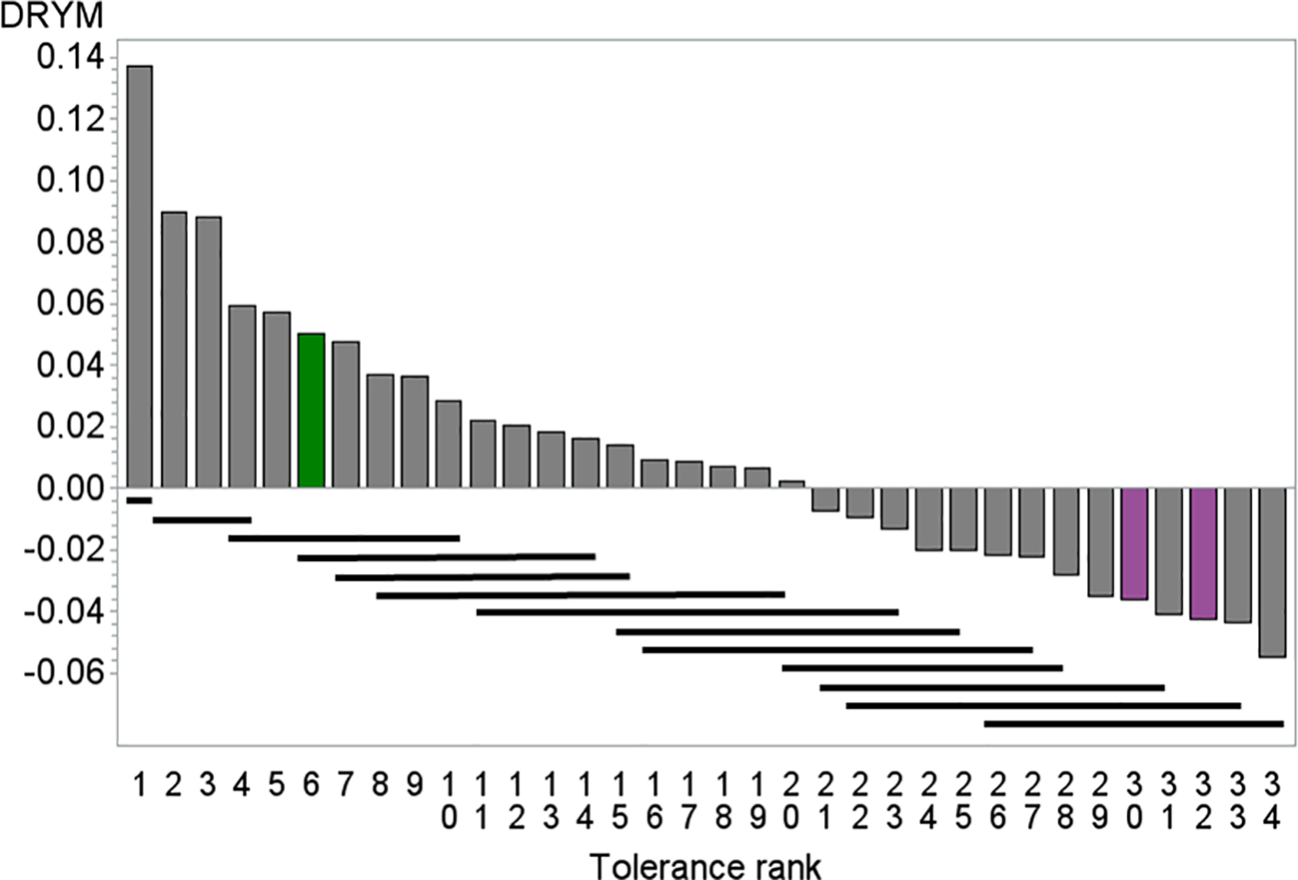
Mean drought tolerance index of 34 potato cultivars depicted against mean tolerance rank, green tolerant cultivar A_t_ (rank = 6), pink sensitive cultivars E_s_ (rank = 32) and R_s_ (rank = 30). Bars indicate significance groups (REGWQ test, alpha = 0.1), cultivars underlined by the same bar are not significant different. Number of replicates 16–22.

F1-Seeds from crosses between tolerant cultivar A_t_ (id 2673) and the sensitive cultivars E_s_ (id 2858) and R_s_ (id 2880) were obtained from the consortium of potato breeders of the GFPi (German Association for the advancement of plant innovation). The parent cultivars were selected from a range of tetraploid cultivars released in Germany, based on the ranking obtained in previous experiments (see **Figure 1**) and the availability of crosses from the breeders (Sprenger et al., 2015). About 800 seeds were germinated on Murashige and Skoog medium with 2% sucrose. The 600 most vigorous plantlets were micro-propagated under axenic conditions to produce vegetative lines. For each line, three cuttings were cultivated under optimal water supply in 3-liter pots in a polytunnel (FGH) at the Max Planck Institute of Molecular Plant Physiology (MPI-MP) for 98 days in 2013 (Pot trial P1) as described before (Sprenger et al., 2015). Substrates and cultivation procedure were as described below. Further details for all trials can be found in **Table 1**. Lines with aberrant shoot or root development, leaf chlorosis or necrosis were excluded. Tubers from the remaining lines were harvested, weighted, sorted by size, and counted. Tubers were subsequently stored at 5°C to be used as seed tubers in the 2014 field trials.

**Table 1:** Experimental design for pot (Trial-id = P), big-bag (Trial-Id = B), and field (Trial-Id = F) trials.

Trial-Id	Culture-Id	Location	T	n	pl	Number of lines	Start date	Flower date	End date	Water (control)	Water (drought)	SI	Thermal sum	cum VPD end
P1		Golm FGH	1	3	1	549								
B2	67199	Golm FGH	2	3	1	227	16.04.2014	10.06.2014	17.07.2014	54.8	29.2	0.49	1306	120.2
P3	68015	JKI Shelter	2	1	2	195	15.05.2014		01.08.2014	20.9	9.6	0.56	1459	209.7
B4	72247	Golm FGH	2	2	3	60	09.04.2015	05.06.2015	19.07.2015	80.9	38.9	0.60	1489	194.0
P5	72292	JKI Shelter	2	4	2	60	12.05.2015	01.07.2015	10.08.2015	22.5	9.9	0.48	1415	215.1
B6	76240	Golm FGH	2	5	1	60	14.04.2016	06.06.2016	17.07.2016	73.9	40.1	0.54	1460	185.7
P7	76354	JKI Shelter	2	4	2	60	09.05.2016	23.06.2016	11.08.2016	20.2	6.3	0.68	1624	195.6
F1	67516	Golm Field	2	1	5	197	22.04.2014	30.06.2014	28.08.2014	89.4	65.6	0.08	1605	148.4
F2	67518	Groß Lüsewitz	2	1	2	191	28.04.2014		27.08.2014	54.3	4.1	0.56	1165	108.6
F3	72275	Golm Field	2	3	5	60	22.04.2015	30.06.2015	17.08.2015	79.7	27.8	0.73	1488	171.4
F4	72396	Groß Lüsewitz	2	2	6	60	28.04.2015	06.07.2015	04.09.2015	77.5	4.5	0.53	1139	113.3
F5	72482	Dethlingen	3	2	16	60	20.04.2015		31.08.2015	78.2	60.5	0.21	1321	94.4
F6	76219	Golm Field	2	3	8	60	21.04.2016	20.06.2016	09.08.2016	60.0	19.4	0.65	1429	139.1
F7	76529	Groß Lüsewitz	2	2	6	60	02.05.2016	29.06.2016	10.08.2016	62.2	6.8	0.70	1041	94.9
F8	76528	Dethlingen	3	2	16	60	19.04.2016		01.09.2016	99.4	70.8	0.18	1412	103.0

Culture Id = experiment reference Id in the MPI-MP database limsdb2 (Köhl et al., 2008). Location: Golm polytunnel (Golm FGH) and Golm field situated in Potsdam-Golm, Germany (52°23’55’’N13°03’56’’E), sandy soil, Julius-Kühn (JKI) Shelter and field Groß Lüsewitz situated in Groß Lüsewitz, Germany (54°04’12’’N12°20’19’’E) and Dethlingen situated in Dethlingen, Germany (52°57’17’’N10°07’33’’E), both sandy loam. T= number of treatment levels: 1 optimal, 2 optimal and drought stress treatment, 3 optimal (50% field capacity), reduced irrigation (30% field capacity), and drought stress (no irrigation). n, number of replicate plots or pots per treatment; pl, number of plants per replicate. Number of lines without parent lines. Planting density 440 plants/100 m². Start date, date of planting into final pot size or field; Flower date, start of flowering in the parent cultivars; End date, date of shoot destruction; Water (control), sum of precipitation and irrigation for control treatment in liter per plant. Irrigation/precipitation volume in the field trials was converted from the area based to a plant based volume by dividing the volume per area by the planting density (4.4 plants/m²). Water (drought), sum of precipitation and irrigation for drought treatment; SI, stress index; Thermal sum in °Cdays, cum vpd end, cumulative VPD at the end of the trial in kPa.

### Microclimate Measurements

Microclimate parameters were measured continuously and logged with a P22 data logger (UP Umweltanalytische Produkte) in the polytunnel and on the managed field sites of the MPI-MP. Air temperature and humidity were measured with HC2-S3 sensors, shielded with a SS3 radiation shield, in 1 min intervals on the field site and 15 min intervals in the polytunnel. Light intensity was measured with a SKP 215 PAR quantum sensor in 1 min intervals. After outlier control, cumulative thermal sums were calculated as the sum of daily thermal sums for each day from the day of planting to haulm destruction. The daily thermal sum (Equation E1) was calculated from the daily minimum (T_min_) and maximum (T_max_) temperature, with a base temperature of 6°C and maximum temperatures above 30°C set to 30°C.

(E1) $$Thermal\;sum=\left(\frac{Tmin+Tmax\left(30{}^{\circ}C\right)}{2}\right)-6$$

The vapor pressure deficit (VPD, in kPa) (E2) was calculated by estimating the saturating vapor pressure (vp_sat_) from the hourly average air temperature T (in °C) and the vapor pressure (vp_air_) from the hourly average relative humidity RH of the air (in %) as follows (Li6400 manual, Licor).

(E2) $$\begin{array}{l} VPD=vp_{sat}-vp_{air}\\ vp_{sat}=\;0.61365*\frac{17.502*T}{240.97+T}\\ vp_{air}=vp_{sat}*\frac{RH}{100} \end{array}$$

The daily midday VPD was calculated as median VPD in the time interval 10–14 MET and summarized as the cumulative VPD from planting date to the actual day. Original data are available at Edal (Köhl, 2018).

### Phenotyping the Segregating Population

Based on the yield data, 225 lines with above-median yield (see results) were selected for preliminary drought tolerance assessment in two container trials (B2, P3) and two field trials (F1, F2) in 2014. For details, see **Table 1**, for field site locations and soil parameters, see heading of **Table 1** and Sprenger et al. (2015), for micrometeorological characterization, see results section “Test environments”.

For the big-bag trial B2, micro-propagated cuttings of each line, of the three parent cultivars and the cultivar Desirée (standard cultivar for experiments at the MPI-MP) were pre-cultivated as described above, transferred to 30-liter big-bags filled with a peat-based potato substrate fertilized with 30 g Novatec classic per bag and cultivated in the polytunnel of the MPI-MP. The design was a randomized split-plot design, with one block for optimal water supply (control) and one block for reduced water supply (stress). Plants were irrigated twice to thrice per week with an injector based line-irrigation system (model CNL 8 l/h, combined with arrow dripper system Cobra-LF, Netafim) to maintain optimal soil water content (40–60% of field capacity). Two weeks after transfer, the water supply to the stress block was reduced to 50% of the volume received by the control block, supplied once a week. In experiment P3, plants were cultivated in a randomized split-plot design at the Julius-Kühn Institute (JKI) in Groß Lüsewitz in 5-liter pots as described before (Sprenger et al., 2015).

For the field trials, tubers were planted manually in a split-plot design on the managed field sites of the MPI-MP in Potsdam-Golm (F1) and of the JKI in Groß Lüsewitz (F2) as described before (Sprenger et al., 2015). In F1, each irrigation regime (control, drought treatment) was represented by one block. Plants were drip-irrigated from the top of the ridge with 10 mm water after sunset when turgor loss was visible at noon (control) or in the morning (drought-treatment). In F2, drought stress was applied by stopping watering at the beginning of emergence.

At the end of each trial, shoots were removed (pot experiments) or killed (field experiments). Tubers were counted and weight, and the starch yield measured as described above. Starch content was determined with a starch balance (Type E6100, MEKU). All phenotyping and yield data are available at Edal (Köhl, 2018).

Tuber production is the relevant response parameter with respect to yield potential and stress tolerance of potato. In contrast to cereal grains, the tuber water content is very high and considerably affected by water supply (Yuan et al., 2003). In a drought stress trial, where soil water content differs between treatments, this can lead to systematically lower tuber water contents and thus underestimate yield in drought-treated plants. As water loss of the tuber increases fresh weight-based starch content, we used a parameter that is less affected by the conditions at harvest by calculating the product of fresh weight and starch content, the starch yield. Starch yield was used accordingly as a response parameter to assess performance. Drought tolerance was estimated by calculating the drought tolerance index DRYM (see Equation E3) on the basis of tuber starch yield values.

Leaf samples were taken from each plant of each line under both treatments (control, stress) in experiment B2 (412 pooled samples from 1,250 single plant samples) and P3 (396 pooled samples) after onset of flowering (Sprenger et al., 2016). In preliminary, yet unpublished studies to the experiments published in Sprenger et al. (2016), different sampling strategies with respect to the developmental stage and the time-course of drought had been compared to find the earliest stage in plant development, in which metabolite and transcript levels would correlate to drought tolerance. These tests had shown that sampling during the flowering time results in better genotype differentiation than earlier sampling dates. Metabolite intensities were measured by gas chromatography-mass spectrometry (Sprenger et al., 2016). Original intensities of each metabolite were normalized to the average original intensity (response) of all annotated analytes in a sample and log_10_-transformed (for data see **Data Sheet 1**). Transcript levels of the 43 genes that are used in the prediction model and the four reference genes were measured by qRT-PCR (Sprenger et al., 2016; Sprenger et al., 2018). The methodology of gene selection is explained in Sprenger et al. (2018). The primer information is given in **Data Sheet 1**. Gene expression values obtained by qRT-PCR measurements are listed in **Data Sheet 1** as 2^−ΔCt^ values after correction for the expression levels of reference genes.

Previous cross-validation of the prediction model had shown that the tolerance was predicted independent of the agro-environment (including water supply), in which the samples were taken (Sprenger et al., 2018). Thus, metabolite and transcript data from both treatments (control and stress) were evaluated together.

### Selection of Subpopulations

From the segregating population, 60 lines were selected in three subpopulations (details see **Figure 1**) based on the tuber starch yield and metabolite/transcript data from three stress trials. Experiment F1 was excluded from drought tolerance analysis as tuber yield was not significantly affected by the stress treatment as a result of high precipitation during the experiment. Lines with low tuber production (<5 tubers per plant under control conditions) or delayed emergence were excluded from the selection.

For the phenotypic subpopulation PP_t_, 20 lines were selected based on the tolerance index DRYM (Sprenger et al., 2015) calculated for each genotype G and experiment E based on the tuber starch yield (SY) as follows:

(E3) $$\begin{array}{l} DRYM_{GxEi}=RelSY_{GxEi}-median\left(relSY_{GxEi}\right)\\ \text{R}elSy_{GXEi}=starchyield{\left(stress\right)}_{GXEI}/\left(starchyield{\left(control\right)}_{GXEI}\right)\\ \text{RelSY}=\;\text{relative}\;\text{starch}\;\text{yield}\\ \text{DRYM}=\text{deviation}\;\text{of}\;\text{relative}\;\text{starch}\;\text{yield}\;\text{from}\;\text{median} \end{array}$$

Within each experiment, lines were ranked according to their DRYM. Lines that ranked among the best 40 lines in at least two experiments were shortlisted for the phenotypic population PP_t_. Lines were removed from this group, when high DRYM values resulted from outliers in tuber yield. Observations were flagged as outliers when the yield value was outside of the mean +/- 3 standard deviations range. The implications of this step are debated in the discussion section “Generation of a segregating population and selection of subpopulations”.

For the selection of the MAS population, MP_t_ and MP_s_, predictive models were generated using a LASSO model using the glmnet package version 4.0 (Friedman et al., 2010) in R version 3.6.3 (R Core Team, 2015). The cv.glmnet function was used to run 10-fold cross-validation. Our model predicted the value of the tolerance index DRYM from a linear combination of metabolite level or transcript level values. The LASSO method was used to achieve a minimum number of predictors by applying the ‘λmin + 1SE’-rule. Thereby, we achieved a sparse subset of 29 metabolites or 23 transcripts for the general linear model to predict the drought tolerance index. This method avoids the risk of overfitting that arises when the number of independent variables is high compared to the number of observations. Missing values in the metabolite (5.5%) and transcript (2.2%) data were estimated by PCA using the Nipals method from the R-package pcaMethods (Stacklies et al., 2007). The training set included 911 samples for metabolite data originating from five independent field experiments and 202 samples for transcript data from three independent field experiments performed in 2011 and 2012 [Supporting information **Tables S6** and **S7** in (Sprenger et al., 2018)]. The resulting models were used to predict the DRYM of 195 lines from metabolite data (806 samples) and transcript data (803 samples). The predicted DRYM values from both models were ranked and averaged to retrieve the 24 most tolerant (MP_t_) and 22 most sensitive (MP_s_) lines. The pedigree and rank of the selected lines is shown in **Data Sheet 1** (**Table S1**). The R-scripts for the development of the LASSO model and the prediction of the tolerance index are available at GitHub https://github.com/HeikeSp/trost_select.

### Characterization of Subpopulations

The 60 selected lines and their parent cultivars were tested for drought tolerance in four 30-liter big-bag (B4, B6) or 5-liter-pot trials (P5, P7) in 2015 and 2016 at the MPI-MP and the JKI, respectively, as described above. Additionally, all lines were cultivated under optimal and reduced water supply in field trials in 2015 and 2016 at three locations. The design was a randomized split-plot design, with three plots per line and treatment at the site Potsdam-Golm (F3, F6) and two plots per line and treatment at the site Groß Lüsewitz (F4, F7). Plants for the drought stress treatment were cultivated under a rain-out-shelter on both sites.

At the location Dethlingen, lines were cultivated in three blocks with either optimal water supply (control), reduced irrigation and without irrigation (stress). In the optimal and the reduced water supply regime, plants were watered with an irrigation boom when soil water content fell below 50 or 30% field capacity, respectively.

Plants were phenotyped for shoot height and phenological stage in the week when the parent cultivars started flowering. The agricultural standard BBCH scale for potato was used for scoring phenological stages.

### Calculation and Statistical Evaluation

Data evaluation was performed in R (3.2.3, RStudio Version 1.0.143, RStudio Inc.; packages knitr, reshape, plyr, dplyr, psych) and in SAS (Version 9.4, SAS Institute). The developmental stages (BBCH) were rank transformed within each experiment, rank = 1 representing the smallest BBCH score. Starch yield (SY) was calculated by multiplying the tuber fresh weight (FW) with the tuber starch content for all tubers harvested from a replicate pot, big-bag, or plot. SY values were processed following the same scheme, both for data in linear as well as logarithmic scale, and normalized with regard to the factors block (B), row (R), and ridge (D). SY values were modelled as a result of a linear effects model associated with the variables B (if two or more blocks were set up in the experiment), R, and D by applying “lm”-function and by treating variables B, R, and D as categorical factors. The obtained model, M, was used to compute normalized SY-values (SY_norm_) using the following “predict”-function, where SYM(B,R,D) are the regressed values of SY based on the obtained linear model, M, and adding the median of the raw values to preserve the absolute magnitude of values before and after normalization.

(E4) $$SY_{norm}=SY-SYM\left(B,\;R,\;D\right)+median\left(SY\right)$$

ANOVA (R-function “aov”) showed no significant association of the factors B, R, and D after normalization of SY-values, indicating successful normalisation.

The DRYM**p** was calculated by normalizing the relative starch yield of each replicate to the median of the three **p**arent cultivars (E5).

(E5) $$DRYMp_{GxEi}=RelSY_{GxEi}-median\left(RelSY_{G=parentEi}\right)$$

The effects of experiment, genotype, subpopulation, treatment, and the interaction between these factors were tested by analysis of variance (ANOVA). Means were compared with TuckeyHSD test and pairwise t-test with Bonferroni p-value adjustment for multiple testing.

For correlation analysis (SAS proc CORR), means of normalized starch yield under control conditions and DRYM**p** were determined for each genotype for the three test systems pot, big-bag, and field.

## Results

### Selection

The segregating population originated from crosses between the tolerant cultivar A_t_ and the sensitive cultivars E_s_ and R_s_. These crosses had been chosen from a set of crosses among 34 potato cultivars that had been previously characterized in drought stress trials [**Figure 1** and (Sprenger et al., 2015)]. Among the crosses available from the breeders, cross A_t_×R_s_ and E_s_×A_t_ were chosen as A_t_ ranked among the six most tolerant lines and was significantly more tolerant than the sensitive parent lines E_s_ and R_s_, which ranked among the five least tolerant lines.

**Figure 2:**
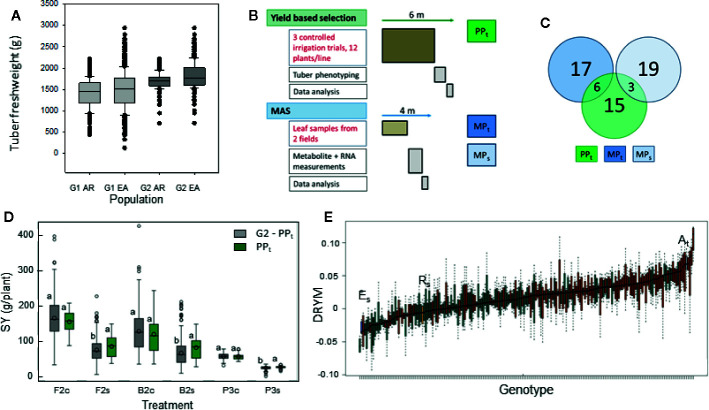
Selection experiment comparing phenotypic selection and MAS from a potato population segregating for drought tolerance. **(A)** Distribution of tuber dry weight in segregating population G1 before selection for above average yield (~600 lines) and population G2 after selection for yield (~200 lines) (experiment P1, yield from three plants, AR = offspring from cross A_t_×R_s_, EA = offspring from cross E_s_×A_t_) **(B)** Scheme comparing procedure and timeline for phenotypic, yield-based selection of subpopulation PP_t_, and MAS of subpopulations MP_t_ selected for superior tolerance and MP_s_ selected for inferior tolerance from 200 G2 lines. Duration of tasked written in red is fixed, duration of those written in black depends on resources. For detailed project plan see **Data Sheet 1**, **Figure S1**. **(C)** Venn diagram showing the number of lines in each subpopulation and the overlap between the subpopulations. There is no overlap between the marker selected populations MP_t_ and MP_s_. **(D)** Tuber starch yield of the phenotypically selected PP_t_ compared to the remaining lines of G2 grown under optimal (c) and reduced (s) water supply in big-bag/pot (B2, P3) and field trials (F2) with significant water stress. Different letters indicate significant differences between population within an experiment and treatment (REGWQ-Test, α = 0.05). **(E)** Drought tolerance predicted from metabolite concentrations for parent cultivars and G2 lines from the crosses A_t_×R_s_ (red) and E_s_×A_t_ (green).

The segregating population obtained from both crosses was reduced to 549 lines (population G1) by the first selection against plants with developmental defects. Tuber number [see data at Edal (Köhl, 2018)] and fresh weight (**Figure 2A**) were higher in the offspring from the cross E_s_×A_t_ than in those from the cross A_t_×R_s_. We selected 200 lines with an above average tuber production [fresh weight and tuber number, population G2 (**Figure 2A**) and (Köhl, 2018)] for drought tolerance assessment in four experiments. One of the experiments was performed in pots, one in big-bags and two under field conditions. In the big-bag system, plants grew in a soil volume similar to that available to a field grown plant and, consequently, produced similar tuber yields. The results from this test environment can thus be expected to be representative of field conditions. Tolerance assessment was based on the starch yield SY under deficit irrigation relative to the starch yield under optimal water supply. Experiment F1 yielded no tolerance data (stress index SI=0.08, no significant effect of treatment, see **Table 1**) because of unusually high precipitation. In the other three experiments, SI ranged between 0.49 and 0.56 with a significant effect of treatment on SY (**Table 1**). Data from these three experiments were used to perform the phenotypic selection based on yield under arid conditions (**Figure 2B**). Yield under arid conditions was normalized by calculating the tolerance index DRYM for each line and each experiment (Equation E3). When calculating this value from a very limited number of replicates per experiment, outliers can result in an extreme DRYM. For example, a very low yield value for a control pot in one of the experiments can result in a very high relative starch yield and thus overestimate the drought tolerance. To obtain a more robust estimate, we used a ranking procedure to select the 24 most drought tolerant lines into subpopulation PP_t_. When comparing the starch yield of PP_t_ with the starch yield of the remaining lines of G2 (G2 minus PP_t_), PP_t_ had a significantly higher starch yield under drought stress, whereas under sufficient water supply, starch yield of PP_t_ war similar to that of the remaining lines of G2 (**Figure 2D**). The theoretical minimum duration of this selection procedure is six months, including four months for plant cultivation until tuber maturity. The cultivation time cannot be shortened by increased investment of labor or money (**Figure 2B**).

For the MAS, two subpopulations MP_t_ and MP_s_ were selected based on the tolerance predicted by the published model (Sprenger et al., 2018) from leaf metabolite (**Figure 2E** and **Data Sheet 1**) and transcript levels (**Data Sheet 1**) measured in two experiments. Lines from the cross A_t_ x R_s_ were overrepresented among lines with a high predicted DRYM. Those lines with the highest and the lowest predicted DRYM were selected into MP_t_ and MP_s_, respectively. As some lines were already selected into PP_t_, additional lines were chosen for MP_t_ and MP_s_ to obtain a total of 60 lines for further testing (**Data Sheet 1**, **Table S1**). As a result, 15 lines were exclusive to PP_t_, six were shared with MP_t_, and three were shared between PP_t_ and MP_s_ (**Figure 2C**) adding up to a total of 24 lines in PP_t_. Ten of the PP_t_ lines originated from the cross E_s_ x A_t_, the remaining 14 lines from the cross A_t_ x R_s_. The lines selected into MP_s_ predominantly originated from the cross E_s_ x A_t_ (21 out of 22 lines). Among the lines selected into MP_t_, the majority (20 out of 24) originated from the cross A_t_ x R_s_ including the six lines shared between PP_t_ and MP_t_ (**Figure 2C**). This overlap was 2.5-fold larger than expected by chance (probability p=0.02; hypergeometric test). The theoretical duration of the MAS procedure is four months with two months being determined by the time the plant requires to reach the optimal sampling stage (BBCH 50–60).

### Test Environments

**Figure 3:**
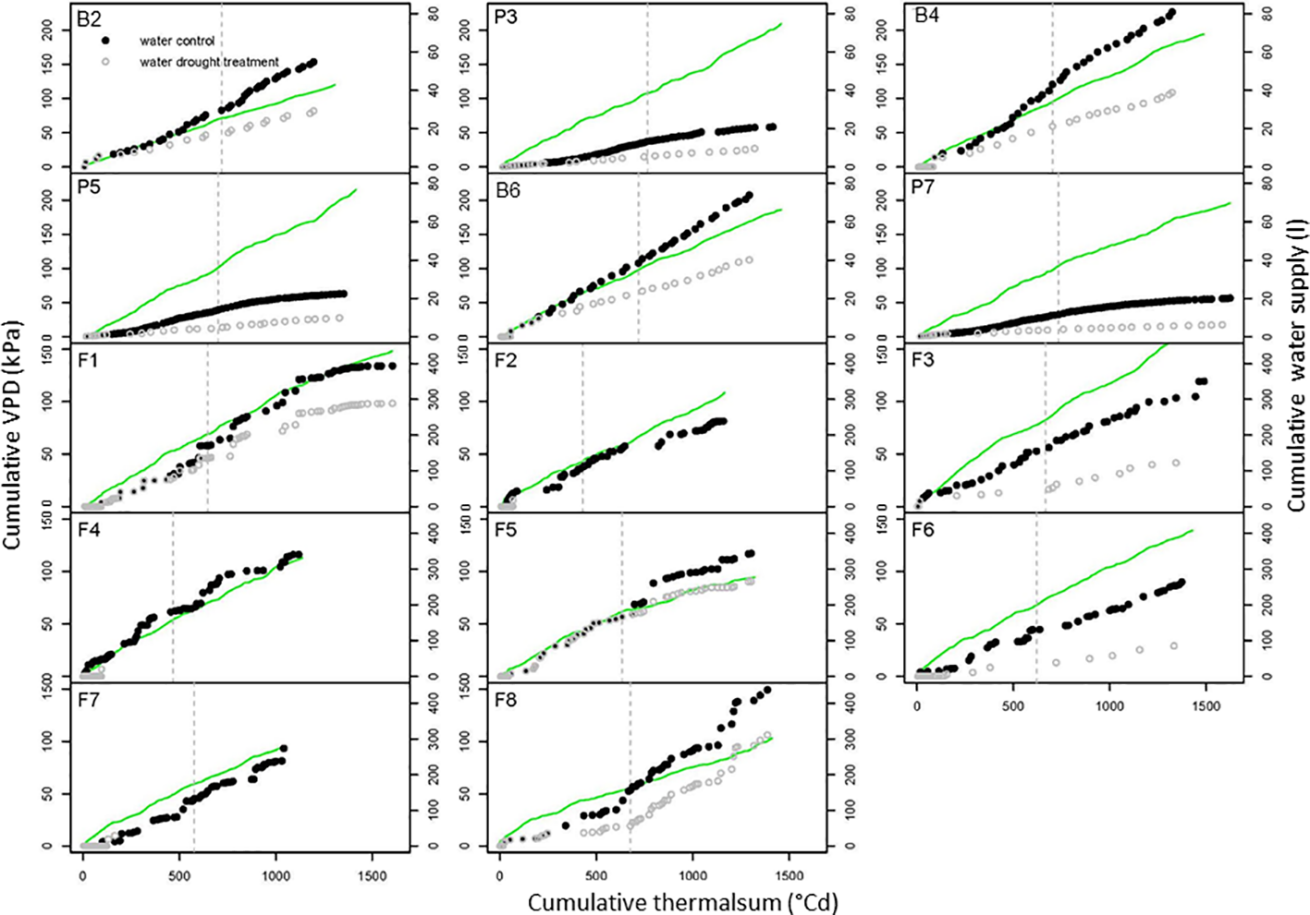
Environmental conditions during drought stress trials. The cumulative vapor pressure deficit (VPD) of the air (green line) and the cumulative water supply to control (closed dots) and drought (open dots) treatments are depicted against the cumulative thermal sum of air temperature for the pot trials P3, P5, and P7, the big-bag trials B2, B4, and B6 and the field trials (F2 to F8). The dotted line indicates the flowering time. Further trial details see **Table 1**.

Stress experiments conducted outside controlled environments require monitoring of environmental condition to allow generalization of the results. Thus, soil water content and micro-meteorological parameters were measured continuously [data set available at Edal (Köhl, 2018)]. The cumulative values of VPD (Equation E2) were plotted against the thermal sum (Equation E1) as a measure of developmental time (**Figure 3**). The duration of the experiments conducted in Golm (B2, B4 and B6, F1, F3, and F6) was controlled to achieve a thermal sum of >1400°Cd at the time of haulm destruction. In the big-bag experiments, the cumulative VPD reached about 200 kPA in all experiments except B2, which was conducted in an untypically wet year with high air humidity.

In the big-bag experiments (B2, B4, and B6), where water was supplied twice a week, the curve of the cumulative water supply followed the cumulative VPD curve, except during the time after flowering, when the water demand of potato is highest during tuber filling. The water supply in the big-bag experiments was much higher than in the experiment P3 and P5, which were conducted in 5-liter pots with correspondingly smaller plants. The amount of water used by plants in big-bags was similar to that of field-grown plants (**Table 1**).

In the field experiments, thermal sums at the end of the experiment were between 1,321 and 1,605°Cd at the sites Golm and Dethlingen, respectively, and around 1,100°Cd at the cooler site Groß Lüsewitz. The cumulative VPD was lower than 150 kPa in most experiments and thus considerably lower than in the pot and big-bag experiments. Cumulative VPD were generally highest at the site Golm, reflecting the higher temperatures and lower air humidity. The water supply to the field resulted from irrigation and precipitation for all control treatments and for the stress treatments in F1, F5, and F8, which were conducted without a shelter. These three experiments had only small differences in water supply between control and stress. Those stress treatments that were conducted under a shelter received less than half of the water supplied to the control treatments. In the experiments F2, F4, and F7, plants had access to a considerable water reservoir in the loamy sand soil (about 60 l m^-2^) in addition to the very low amount of water supplied by irrigation.

### Growth and Development

**Figure 4:**
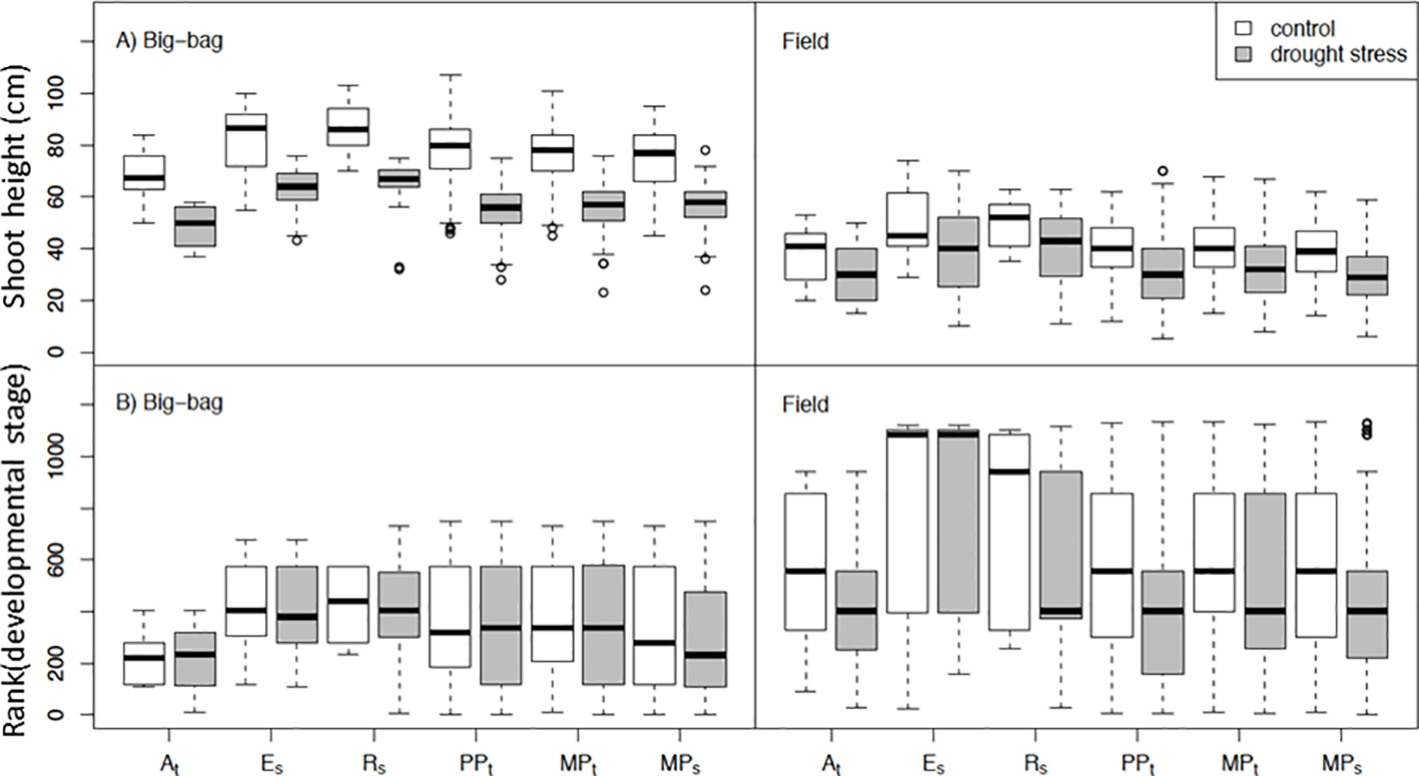
Shoot height and development of subpopulations under control conditions and drought stress. Distribution of shoot height **(A)** and rank-transformed developmental stage **(B)** in subpopulations PP_t_, MP_t_ and MP_s_ and parents A_t_, E_s_ and R_s_ under control and drought treatment in two big-bag and two field trials. For the results of ANOVA see **Table 2**.

Shoot height and developmental stage were phenotyped when the parent cultivars A_t_, E_s_, and R_s_ started flowering (**Figure 4** and **Table 2** for statistics). The parent genotypes showed the developmental characteristics observed for these cultivars in earlier field trials. The tolerant cultivar A_t_ had shorter shoots and flowered earlier than the sensitive cultivars E_s_ and R_s_. The shoot height of the three subpopulations was intermediate between the parents with no obvious differences among them. Drought treatment decreased shoot height significantly (p < 0.05) in all experiments except F1 (**Table 2**). However, there was only weak interaction between treatment and genotype effects on shoot height and development. Drought stress affected development less consistently than shoot height. Drought stressed plants delayed flowering in several experiments, however, earlier flowering was also observed (P4, F6). The lack of significant interaction effects suggests no significant genetic variation in the response of shoot growth and development to drought stress.

**Table 2:** ANOVA on the effect of treatment (E), subpopulation (G), and the treatment × subpopulation interaction (E×G) on shoot height and development in big-bag and field trials.

Trial-Id	Parameter	Df	F(E)	p(E)	F(G)	p (G)	F(E×G)	p(E×G)
B2	Height	322	228.496	<0.0001	2.055	0.130	3.994	0.0193
B4	Height	710	1412.484	<0.0001	3.273	0.038	8.118	0.0003
B6	Height	713	1303.641	<0.0001	0.532	0.587	1.115	0.3280
F1	Height	349	0.287	0.5920	1.029	0.358	1.995	0.1380
F3	Height	1045	696.213	<0.0001	4.49	0.011	2.946	0.0530
F6	Height	1025	495.502	<0.0001	18.766	<0.0001	0.552	0.5760
B2	BBCH	322	9.656	0.0021	0.876	0.417	0.517	0.5966
B4	BBCH	710	6.829	0.0092	13.449	<0.0001	1.128	0.3244
B6	BBCH	713	5.157	0.0234	3.796	0.0229	2.868	0.0574
F1	BBCH	349	0.01	0.9204	0.567	0.5676	2.537	0.0805
F3	BBCH	1052	63.423	<0.0001	13.237	<0.0001	0.149	0.8620
F6	BBCH	1038	45.478	<0.0001	4.103	0.0168	1.800	0.1658

The shoot height was normalized to the median shoot height of the respective experiment. The developmental stage (BBCH) was rank-transformed (rank 1 least developed) within each experiment. The trial-id of big-bag experiments starts with a B, of field experiments with a F. Df, degrees of freedom for the model error; F, F statistic; p, probability. Df(E)= 1, Df(G) = 2, Df(E×G) = 2.

### Yield

Tuber fresh weight, starch content, tuber numbers, and their size distribution were analyzed as indicators for yield quality and allocation patterns of the different lines. Tubers were fractionated into the “marketable” medium size fraction (35 to 60 mm), a small and an oversized fraction (**Data Sheet 1**, **Figure S2**). The total tuber number under control conditions was similar in PP_t_ and MP_s_ and slightly higher in MP_t_. Size distribution was similar in all subpopulations with a slight trend towards less oversized and a higher number of small tubers in MP_t_. Drought stress significantly (p < 0.05) reduced the number of oversized and medium-sized tubers in all populations, whereas the number of small tubers remained constant.

**Figure 5:**
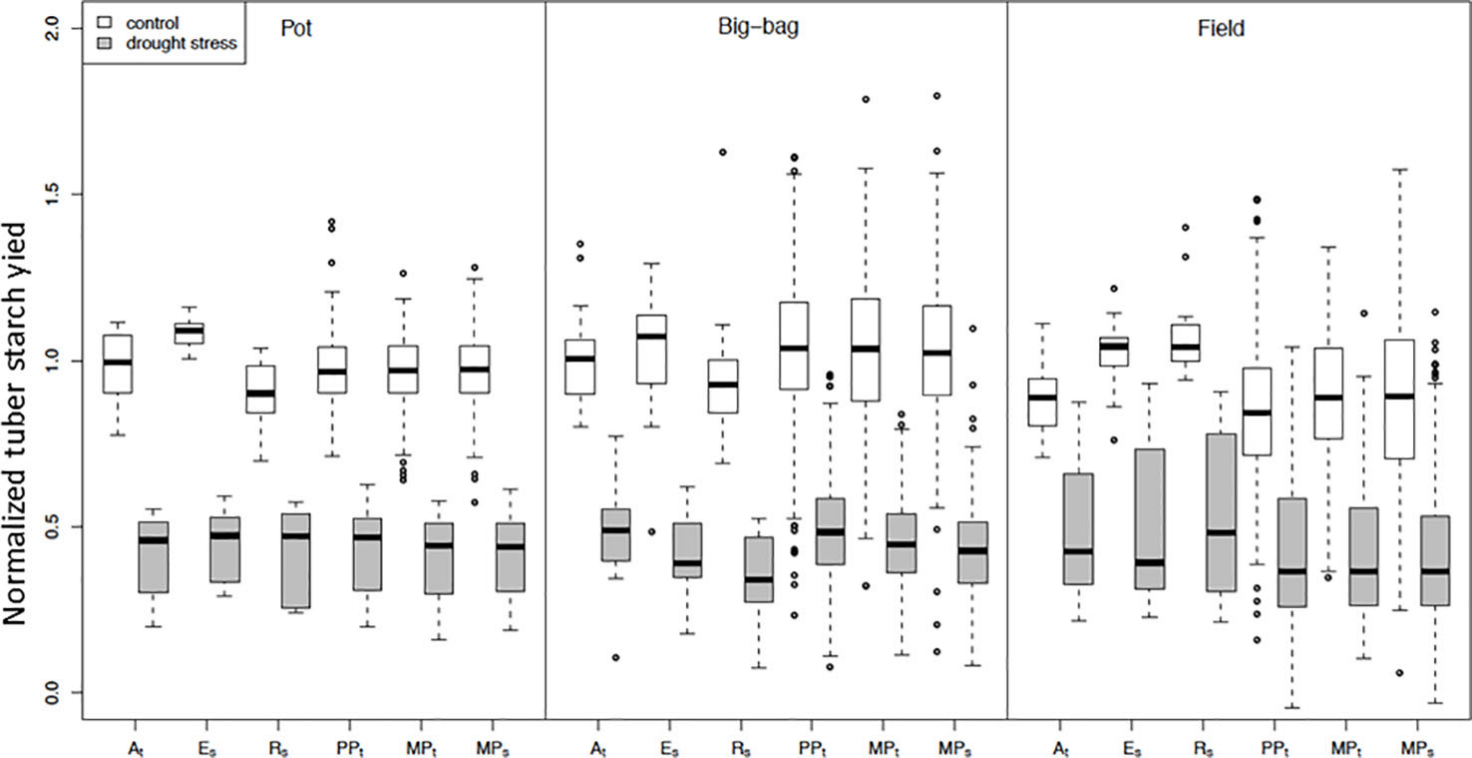
Normalized tuber starch yield of subpopulations PP_t_, MP_t_ and MP_s_ and parent cultivars A_t_, E_s_ and R_s_ under control conditions and drought stress. Distribution of normalized mean tuber starch yield under control and stress treatments in pot (P3, P5, P7), big-bag (B2, B4, B6), and field trials (without F1). Starch yield was corrected for spatial effects and normalized to the median of the starch yield of the parent cultivars under control conditions. Result of ANOVA on starch yield in single experiments see **Table 3**.

As a parameter for yield, we analyzed SY (**Figure 5**), which is the product of starch content and tuber fresh weight and equivalent to the dry matter of the tuber. To permit the integration of several experiments, tuber starch yield was normalized to the median tuber starch yield of the parent cultivars grown under optimal water supply. The experiment-wise ANOVA for the effects of drought treatment (E), subpopulation (G), and their interaction (G×E) on normalized starch yield (**Table 3**) indicated a significant treatment effect for all experiments except F1. In most pot and big-bag experiments, normalized starch yield was significantly affected by either G or G×E. In the field experiments, genotype and interaction effects were too small to be significant. To increase the statistical power, we calculated the average normalized starch yield for all lines and the parents for the test systems pot, big-bag and field (**Figure 5**). Under optimal water supply, average tuber starch yield of the subpopulations was similar to that of the three parent cultivars in pot and big-bag experiments, as indicated by the mean value around 1. Under field conditions, the average starch yield of the two sensitive parents E_s_ and R_s_ was higher than that of the three subpopulations. Drought treatment reduced normalized starch yield to less than 0.5. In pot experiments, average SY under stress was similar in all three subpopulations. In big-bag trials, average stress SY was highest in PP_t_, intermediate in MP_t_ and lowest in MP_s_. ANOVA on the normalized starch yield of the subpopulations (**Table 3**) indicated significant G and G×E effects for the big-bag trials and weakly significant G and G×E effects for the field trials. In the big-bag and field experiments, the highest SY measured under stress conditions in PP_t_, MP_t_ and MP_s_ lines exceeded the highest values measured for the three parent cultivars, suggesting that these populations may contain genotypes with superior drought tolerance, warranting a detailed analysis of the lines.

**Table 3:** ANOVA on the effect of treatment (E), subpopulation (G), and the treatment x subpopulation interaction (E×G) on normalized tuber starch yield in pot, big-bag and field trials.

Trial-Id	Df	F(E)	p(E)	F(G)	p (G)	F(E×G)	p(E×G)
B2	308	223.822	**<0.0001**	0.34	0.7123	7.892	**0.0005**
P3	231	2105.94	**<0.0001**	1.51	0.2232	5.408	**0.0051**
B4	703	2940.21	**<0.0001**	4.776	**0.0087**	0.249	0.7798
P5	699	8820.39	**<0.0001**	0.933	0.3940	3.153	**0.0433**
B6	705	1842.26	**<0.0001**	6.585	**0.0015**	1.395	0.2486
P7	676	11372.3	**<0.0001**	2.306	0.1000	0.817	0.4420
F1	114	1.917	0.169	0.455	0.6350	0.181	0.8350
F2	227	346.7	**<0.0001**	0.224	0.8000	1.483	0.2290
F3	341	1553.22	**<0.0001**	7.071	**0.0010**	4.635	**0.0103**
F4	221	365.873	**<0.0001**	0.438	0.6460	0.054	0.9480
F5	220	35.313	**<0.0001**	0.184	0.8320	0.579	0.5610
F6	351	1924.41	**<0.0001**	1.725	0.1800	0.919	0.4000
F7	211	1012.94	**<0.0001**	0.452	0.6370	0.624	0.5370
F8	216	43.889	**<0.0001**	3.319	**0.0380**	0.266	0.7670

Df, degrees of freedom for the model error; F, F statistic; p, probability (in bold when p < 0.05).

### Drought Tolerance

Drought tolerance was assessed by the deviation of the relative starch yield from the median relative starch yield of the three parent cultivars DRYM**p** (see Material and Methods, Equation E4). Mean DRYM**p** were calculated for each genotype and each experiment and then summarized within the groups parent cultivars and the three subpopulations PP_t_, MP_t_, and MP_s_. **Figure S3** (**Data Sheet 1**) depicts the distribution of DRYM**p** for these groups separately for the three test environments pot, big-bag, and field. **Data Sheet 1** **Table S2** (**Data Sheet 1**) lists the descriptive statistics. In all environments, variation of DRYM**p**, estimated by the standard deviation, was highest in the segregating population G2 and lowest in the parent cultivars. The standard deviation of DRYM**p** was substantially smaller in the pot experiments than in the big-bag and field experiments. In pot experiments, mean DRYM**p** of the segregating population G2 was lower than that of the parent cultivars. In contrast, mean and median DRYM**p** of G2 were higher than those of the parent cultivars in big-bag and field experiments, which suggests that the crosses may have yielded genotypes of superior drought tolerance compared to the parent cultivars. Analysis of variance on the data set containing the segregating population and the selected subpopulations revealed a significant effect of the population on DRYM**p** in all three test environments (**Table 4**). When comparing the selected populations, PP_t_ had the highest mean DRYM**p** in pot, big-bag, and field experiments. The MAS populations MP_t_ and MP_s_ had lower mean DRYM**p**, which were not significantly different from each other. In all three test environments, the selected populations contained genotypes with a superior DRYM**p** higher than the percentile 90 of the parent cultivars. We therefore analyzed the drought tolerance of each line in relationship to its yield potential.

**Table 4:** Result of an analysis of covariance on the effect of population and the covariate cumulative VPD (cum_vpd) on drought tolerance DRYM**p**.

Model	Type	Source	df	F	p
1	pot	error	1096		
1	pot	population	6	8.3	<0.0001
1	pot	cum_vpd	1	48.1	<0.0001
1	big-bag	error	1207		
1	big-bag	population	6	3.9	0.0007
1	big-bag	cum_vpd	1	28.0	<0.0001
1	field	error	1195		
1	field	population	6	2.4	0.0265
1	field	cum_vpd	1	12.2	0.0005
2	pot	error	1057		
2	pot	population	3	13.2	<0.0001
2	pot	cum_vpd	1	51.1	<0.0001
2	big-bag	error	1156		
2	big-bag	population	3	5.7	0.0007
2	big-bag	cum_vpd	1	29.7	<0.0001
2	field	error	1151		
2	field	population	3	3.8	0.0106
2	field	cum_vpd	1	12.8	0.0004

In Model 1, the factor population contains the three subpopulations, the parents, and the population G2 without the lines that were selected into the subpopulations. Model 2 does not contain the data on the parents.

### Relationship Between Yield Potential and Drought Tolerance

**Figure 6:**
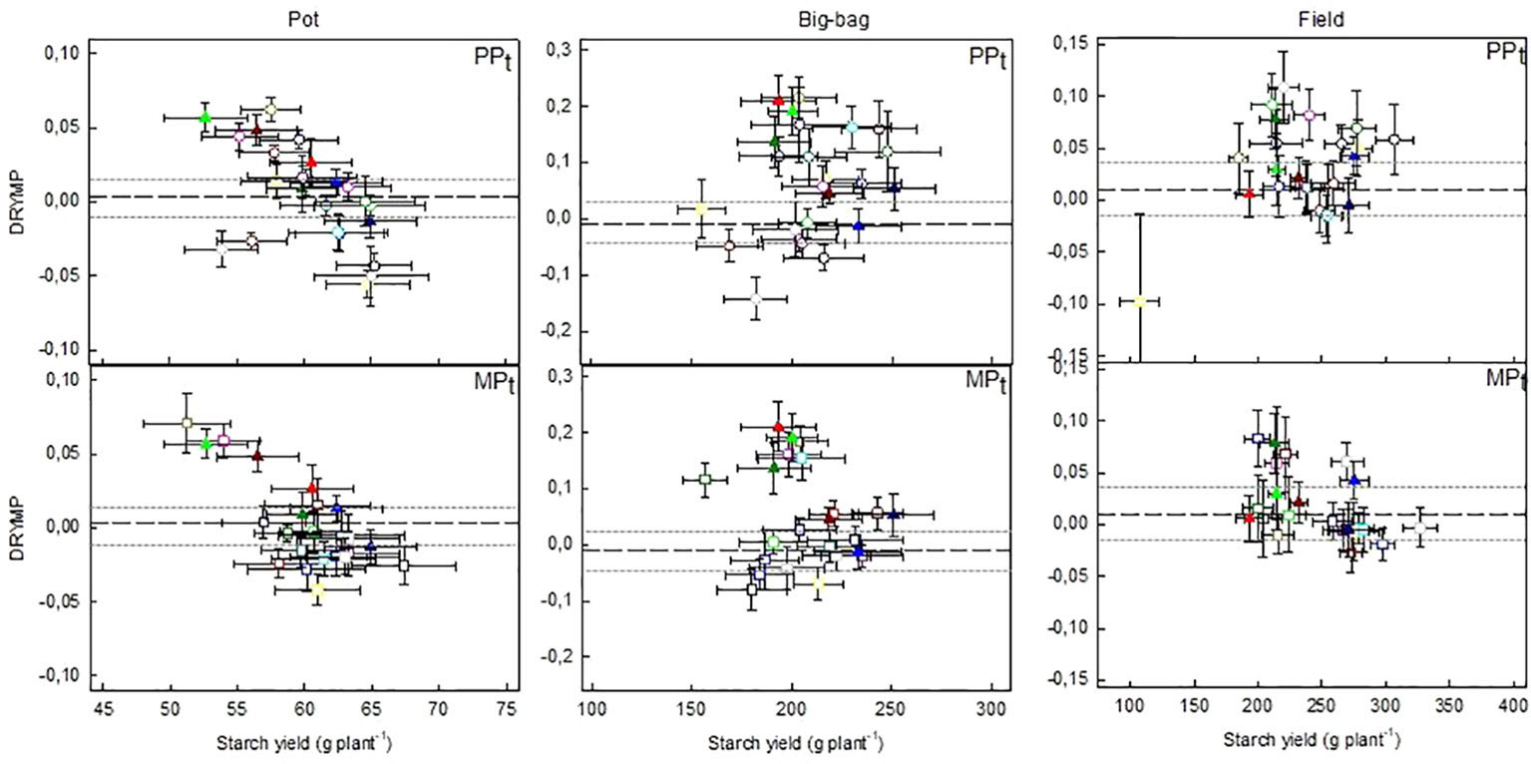
Relationship between drought tolerance (DRYM**p**) and starch yield under optimal water supply in lines selected for superior drought tolerance. Mean and standard error (SE) of DRYM**p** depicted against mean and SE of tuber starch yield of the lines in PP_t_ and MP_t_ in pot, big-bag and field trials. Tuber starch yield was normalized to account for the spatial effects in the experiments. The reference lines indicate the mean (dashed line) and 95% confidence interval (dotted line) of DRYM**p** in the respective test system. Lines common in PP_t_ and MP_t_ are represented by closed triangles, including three lines with superior performance: AR185 (light green triangle), AR196 (dark green triangle), and AR121 (light red triangle). The outlier in PP_t_, field is line AR21. Number of pot trials 3, big-bag trials 3, field trials 7. Data for the lines of MP_s_ are shown in **Data Sheet 1**, **Figure S4**.

The relationship between tolerance and yield is depicted in **Figure 6** (PP_t_ and MP_t_) and **Figure S4** (MP_s_) for the three test systems. For each line of the three subpopulations, average and standard error of DRYM**p** are plotted against the average and standard error of tuber starch yield under optimal water supply. The median starch yield of the 60 lines was 60, 204, or 240 g plant^-1^ in the pot, big-bag or field experiments, respectively. In all three subpopulations about 50% of the lines had a starch yield above the median starch yield, indicating that the selection did not favor high- or low-yielding lines in any of the populations independent of the test system. The starch yields in the pot system correlated weakly with the starch yield in the big-bag system and the field system (**Table 5**). Furthermore, the correlation between the starch yield in the big-bag and the field system were too weak to be significant (**Table 5**). This suggests a considerable interaction between genotype and the test system on starch yield. The correlation between starch yield and DRYM**p** was significantly negative in the pot and field trials and not significant in the big-bag trials (**Table 5**).

**Table 5:** Result of Pearson correlation analysis between drought tolerance DRYMp and normalized tuber starch yield under optimal water supply (SY) in pot (P), big-bag (B), and field (F) test environments.

Parameter	DRYMp(P)	SY(P)	DRYMp(B)	SY(B)	DRYMp(F)	SY(F)
DRYM**p(P)**	1	0.0001	0.0001	0.7234	0.8441	0.0001
**SY(P)**	**-0.6619**	1	0.0228	0.0444	0.2293	0.0112
DRYM**p(B)**	**0.5859**	-0.2961	1	0.4044	0.3826	0.0012
**SY(B)**	0.0471	0.2627	0.1106	1	0.3399	0.1731
DRYM**p(F)**	0.0262	0.1589	0.1158	-0.126	1	0.0054
**SY(F)**	**-0.5044**	**0.3279**	**-0.4112**	0.18	**-0.3577**	1

Cells below and left of the diagonal contain the correlation coefficients (bold print indicates p < 0.05), cells above the diagonal p-values. N = 59 (outlier line 899484 excluded).

The DRYM**p** was significantly correlated between pot and big-bag experiments (Pearson correlation, p < 0.0001), but there was no correlation between the DRYM**p** found in field environments and those in pot and big-bag systems. The number of lines with a DRYM**p** above the confidence interval of the parent’s DRYM**p** was 8 (pot), 15 (big-bag), and 11 (field) out of 24 lines in population PP_t._ In population MP_t_, DRYM**p** was above the parent’s confidence interval in 6 (pot), 12 (big-bag), or 6 (field) lines. Two of the six lines that were selected in both populations PP_t_ and MP_t_ had very high drought tolerance (mean above 0.025, rank above 55) in pot and big-bag trials. One of the lines selected into PP_t_ and MP_t_. was among the four most tolerant lines under field conditions. Thus, both selection methods identified lines of superior drought tolerance.

## Discussion

Breeding for complex, polygenic traits like yield or drought tolerance (Slater et al., 2014a) based on direct selection for yield requires multi-year, multi-environment field studies on large populations. In consequence, it is slow and expensive. The rapidity of environmental changes predicted by global climate change models requires more efficient breeding. MAS for complex traits has been shown to be rapid and precise when based on large effect QTL, but less successful for polygenic traits without large effect QTL such as yield (Slater et al., 2016). When comparing genomic selection based on SNP markers to phenotypic selection in a limited number of trials on barley, both methods performed equally well in selection for Fusarium head blight resistance, but failed to increase yield significantly (Sallam and Smith, 2016). As an alternative to genomic selection, we suggested a metabolite and transcript marker-based model that predicts drought tolerance with an accuracy of >90% independent of the agro-environment (Sprenger et al., 2018). In the present study, we compared the performance of a selection based on this model to a phenotypic selection based on yield data from a limited set of drought trials.

### Generation of a Segregating Population and Selection of Subpopulations

The comparison of selection methods requires a population of genotypes that segregate for the parameter of interest. For that purpose, we chose crosses between one tolerant cultivar (A_t_) and two sensitive cultivars (E_s_, R_s_) based on the cultivar tolerance data gained before in multi-year, multi-environment field trials (Sprenger et al., 2015).

In most crop species, segregating populations are generated by crossing parents of contrasting tolerance and then producing offspring by selfing or by the production of double-haploid lines. As potato is a clonally propagated crop, each seedling yields a vegetative line, which can be maintained *in vitro*. However, as a result of its outbreeding nature, potato is highly heterozygous (Slater et al., 2014a; Gopal, 2015). The narrow genetic base of modern *Solanum tuberosum* cultivars results in acute inbreeding depression (Slater et al., 2014a; Gopal, 2015). Crosses thus produce a high percentage of offspring with inferior performance. Accordingly, the initial seedling population contained a substantial percentage of plants with visual defects or inferior tuber production. Extremely slow growing and low yielding plants may be mistaken for tolerant plants, as they consume less water, thus experiencing higher soil humidity. To avoid this confounding effect, we selected those 200 lines for further characterization that showed tuber yields in the same range as the parent cultivars. The percentage of retained seedlings was within the range of 20 to 50% recommended (Gopal, 2015).

For the selection of the subpopulations, 200 lines from population G2 were cultivated in four drought stress trials. The yield reduction under drought in the three experiments that delivered the data for selection into population PP_t_ was around 50% and thus is comparable to a selection in an arid agricultural environment. Selection under these conditions is generally selection for maintenance of yield, not for survival. For the MAS populations, the 24 most tolerant and the 22 least tolerant lines were selected into MP_t_ and MP_s_, respectively, based on the tolerance predicted by the LASSO model from transcript and metabolite levels in leaves from two experiments. As some lines selected in PP_t_ had also been selected in MP_t_ (6) or MP_s_ (3), the total number of lines in all three populations was 60. The overlap between the two subpopulations PP_t_. and MP_t_, selected for superior tolerance was higher than chance.

The percentage of lines selected into a tolerant subpopulation from the segregating G2 population is high (22 to 24 out of 200), resulting in a low selection intensity compared to the intensity applied by breeders. Furthermore, the size of the selected subpopulations is small. While the population size was sufficient to estimate and test for differences in population means, the test power was low when applying contingency table statistics to find out, whether PP_t_ contains a different number of tolerant lines compared to MP_t_. The limiting factors in the selection experiment were the area of the shelters available for the first characterization of the 200 lines and the subsequent phenotyping of the selected populations, the maximum duration of the funding period and the maximum amount of fundable work force. We operated a compromise between the number of replicates per line and the number of lines per experiments based on previous experience with drought tolerance trials in potato (Sprenger et al., 2015; Rudack et al., 2017) and performed joint evaluation of yield and tolerance data from three partners to increase statistical power.

### Drought Tolerance Determination in Multi-Environment Trials

Drought tolerance determination in potato in controlled environment pot trials is highly reproducible, but correlates weakly with drought tolerance under field conditions (**Table 5** and (Sprenger et al., 2015). Traits like yield, starch content and drought tolerance show large genotype × environment interaction (Anithakumari, 2011; Slater et al., 2014b; Gopal, 2015). In consequence, traits having beneficial effects on yield in one drought-stress pattern do not necessarily have beneficial effect in another drought stress scenario. This means that the characterization of drought stress experiments by key environmental metadata is a prerequisite for the replication of experiments in phenotyping platforms or managed field trials and the comparison between trials. Furthermore, these metadata are required for the subsequent generalization of test results to other target environments and the use of the data in modelling approaches using climate change scenarios (Millet et al., 2016).

The three test systems pot, big-bag, and field differed in the size of the soil water capacity available to the plant. In pots, plants rely entirely on the daily water supply as the small substrate volume buffers but a very small amount of water available to the plant. The plant available water is defined by two plant – soil parameters. The permanent wilting point is the lower limit. The upper limit is the maximal soil water content that does not cause damage due to water logging. In the field, however, roots can extract water from a much larger soil volume. This allows to buffer a much larger volume of water. In soils with a large water capacity as in experiments F2, F4, and F7, this permits to maintain the stressed plants on the water stored in the soil without the need for further irrigation. Under these conditions, root parameters, especially rooting depth, affect the amount of water available to a genotype (Puertolas et al., 2014). While in a situation, where the plants depend on the soil water storage, deep rooting enhances performance under drought, this will not be the case in pot grown plants, where water supply rather than the stored water determines the amount of water available to the plant. This may be one of the reasons for the low correlation between drought tolerance determined in pot experiments and field experiments. The correlation was only marginally better when the soil volume was increased by performing the experiment in big-bags.

To characterize the test environments, we used an approach published for maize (Chenu et al., 2011). Maize target production environments were classified based on the relationship between crop water status calculated as water supply/demand ratio versus thermal time pre- and post-flowering. We followed this approach by depicting water supply and the parameter VPD, which governs water demand, against thermal time. The drought stress treatments in the pot and big-bag trials mainly represented the early-terminal (ET) stress pattern, in which drought stress begins early in development and persists until harvests. Stress increases more gradually in pot or big-bag trials with deficit irrigation than in the stress type ET described for European maize environments, where stress increases steeply for the first 400°Cd (Harrison et al., 2014). The gradual increase pattern is found in the field trials conducted under a shelter with deficit irrigation (F3, F6). In contrast, stress increased steeply in those field trials that were conducted under a shelter without further irrigation (F2, F4, F6). However, the stress was mitigated by the comparatively low cumulative VPD at the site of these experiments. The field experiments F5 and F8, which were conducted without a shelter, represent mild late-terminal stress (F5) and late-relieved stress (F8). These stress patterns are typical for the subcontinental part of central Europe, where early terminal stress, late terminal stress and early relieved stress appeared in around 5% of the maize cultivations between 1975 and 2010 (Harrison et al., 2014). In conclusion, the key environmental metadata of our trials suggest that these trials represent common drought stress scenarios in the drought-prone agro-environments of Central Europe.

### Drought Tolerance Index

The comparison of drought tolerance information from different trials under non-standardized conditions requires data normalization to account for differences in the degree of stress. Various tolerance indices have been suggested for this purpose, e.g. the stress susceptibility index SSI, the stress tolerance index STI and DRYM (Fischer and Maurer, 1978; Fernandez, 1992; Sprenger et al., 2015). Tests on artificial data sets have shown a superior performance of DRYM with respect to the detection of lines with high tolerance, independent of their yield potential (Sprenger et al., 2015). However, the normalization of DRYM to the median of the relative starch yield of all genotypes means that the relative position of the DRYM changes with the composition of the population. When selecting a population with a higher percentage of tolerant lines, the absolute value of the median will increase and selection for high tolerance will not result in increased DRYM values compared to the original population. This problem also affects the other indices. When the percentage of tolerant lines in a population increases, this will result in a decreased SI under constant stress conditions, thus shifting the median of the SSI and the STI. This is a common problem when comparing populations during selection, which can be solved by using check genotypes that are included in each trial as a basis for normalization. For the determination of drought tolerance, we therefore calculated the DRYM**p**, which was normalized to the median of the parent cultivars, which were used as check cultivars on all sites in this study (see Equation E4). The main disadvantage of this approach is the lower precision of the estimate as the median of the parent cultivars is calculated from a smaller data set than the median of the population. This can be compensated for by increasing the number of replicates for the parent cultivars compared to the tested lines.

### Side Effects of Selection

The offspring of crosses may show a wide variation in development, morphology, and yield potential. Developmental variation may interfere with the assessment of drought tolerance, when the developmental stage that is most sensitive to insufficient water supply is reached at different times by different genotypes. In late-terminal stress scenarios, earliness is a trait that allows the crop to avoid water limitations in sensitive intervals such as the anthesis-silking interval in maize and the time between panicle development and anthesis in rice (Fukai and Cooper, 1995; Harrison et al., 2014). In potato, however, there is no consensus as to the timings of the most sensitive period (Carli et al., 2014; Sprenger et al., 2015). Nevertheless, the potential interaction between development and drought tolerance and between growth and drought tolerance requires testing for side effects of selection. We therefore checked whether selection affected development, tuber size distribution, or yield potential. The latter was estimated from the tuber starch yield under optimal water supply. The results indicated that growth and development of the selected genotypes were in the range of the parent cultivars under optimal water supply. There was no effect of selection on tuber starch yield in the pot and big-bag trials. However, under optimal water supply, parent cultivars were superior in starch yield under field conditions as expected for cultivars that an expert breeder selected for optimal starch yield in field production.

### Phenotypic Selection Versus MAS

Estimating drought tolerance based on DRYM**p** for parent cultivars, segregating population G2, and selected subpopulations, we found that all three subpopulations contained lines that were more tolerant than the parent cultivars. Negative correlations between tolerance and tuber starch yield under optimal water supply confirmed the risk of a yield penalty in drought-tolerant potato genotypes, which has been found before in a study on potato cultivars (Sprenger et al., 2015). Generally, high productivity is apparently rarely combined with high stress resilience (Thiry et al., 2016). In rice, yield penalty of drought tolerance results from a linkage between the QTL QDTY1.1 for grain yield under drought with the green revolution gene *sd1*, which was selected against when semi-dwarf types were bred (Vikram et al., 2016). The breeders are thus most interested to know whether the selected populations contained lines with superior drought tolerance and medium to high yield indicating that both selection methods allow identifying lines, which are tolerant without incurring a yield penalty. We thus compared the yield of highly tolerant lines in the subpopulations to the interquartile range of starch yields found in cultivar trials on the test site of the MPI-MP (Sprenger et al., 2015), which was 196–278 g plant^-1^. In lines AR185 or AR196, which were selected in both, PP_t_ and MP_t_, the starch yield under optimal water supply was 210 or 215 g plant^-1^, respectively, under field conditions.

The phenotypically selected population PP_t_ had a significantly higher average DRYM**p** than the parent cultivars. The average DRYM**p** was lower in MP_t_ and MP_s_, which were not significantly different from each other. All three populations contained lines with superior drought tolerance, many of which were selected in both PP_t_ and one of the MAS populations. The surprise was that the DRYM**p** of the two MAS populations selected for superior (MP_t_) or inferior (MP_s_) tolerance were not significantly different from each other and both populations contained highly tolerant lines. This unexpected result suggests that our marker model allows selecting for high tolerance, but not against low tolerance. This is a clear limitation of the approach as it does prevent the use of the model to select against drought-sensitive lines during breeding. This result is unexpected as during model validation, both sensitive and tolerant cultivars were classified correctly independent of the agro-environment (Sprenger et al., 2018). The main difference between the population in Sprenger et al. (2018) and this population is its genetic composition. The reliability of genomic prediction decreases when the genetic composition of the population changes (de Roos et al., 2009). It is known that epistatic interactions can skew the evaluation of QTL effects and thus bias the selection procedure (Ribaut and Hoisington, 1998). Our interpretation is that there are several tolerance mechanisms in potato and that additional traits not detected by our marker model became effective when genetic material was recombined. These additional mechanisms could be identified by closer investigation of those lines, in which the observed DRYM**p** is significantly higher than the predicted tolerance level.

The rate-limiting factor in selection is the developmental stage, at which samples can be taken. The time depends on plant development and thus cannot be sped up by increased investments. Yield-based selection requires a complete growth cycle, which is about four months in potato. Leaf samples for the metabolite and transcript based selection can be taken much earlier, ideally, as previous investigations have shown, during flowering. The minimum cultivation time is thus 1.5–2 months. The time required for the subsequent sample analysis depends on the investment in personnel and machinery and can thus be accelerated as needed. As the prediction is independent of the agro-environment (Sprenger et al., 2018), no drought stress trial is required for the metabolite/transcript based selection. Samples could thus be taken from plants grown on farmer’s field, taking away the need for managed drought trials in selection. In contrast to yield-based selection, which requires several trials to obtain a reliable estimate, samples from less than five plants from a single field already provide a good prediction for metabolite/transcript based selection (Sprenger et al., 2018). This factor allows increasing the number of tested lines substantially compared to a classic selection based on drought stress trials. Thus, our metabolite/transcript model approach allows the breeder to test more lines in a shorter time compared to yield-based selection in arid environments. The main weakness of the model is the failure to select against drought-sensitive genotypes. Thus, after using the model to select the most tolerant 10% of a segregating population, further selection methods are required to remove sensitive genotypes. In crops like potato, where genomic selection is available, genomic selection during the seedling stage of potato breeding could precede the metabolite-model based selection and remove sensitive genotypes. Phenotypic selection on secondary traits or participatory breeding approaches (Atlin et al., 2001; Lafitte et al., 2003) could follow the transcript/metabolite model based selection in orphan crops.

In conclusion, our selection experiment on a segregating potato population showed that the selection for drought tolerance based on a transcript and metabolite marker model seems to be an efficient alternative to phenotypic selection based on yield measurements in drought-stress trials. As the metabolite/transcript model selection is limited to selection for tolerance, while failing to select against sensitivity, it does not provide a stand-alone solution but may work best in combination with genomic or phenotypic selection.

## Data Availability Statement

The data are available at the sources cited in the article, at EDAL, Github, and as **Supplementary Material**.

## Author Contributions

MH and KK conducted selection and tolerance experiments, evaluated the data, produced the figures, and wrote the manuscript. HS, EZ, DH and JK measured and evaluated the metabolite and transcript levels and designed the prediction model, SS and RP conducted tolerance experiments, DW normalized the starch yield data, SS, RP, DW, EZ, DH, JK, and KK designed the study and supervised the work.

## Funding

The work was funded by the Fachagentur Nachwachsende Rohstoffe (grant 22010913) and the Gemeinschaft zur Förderung der privaten deutschen Pflanzenzüchtung (GFPi). We thank the breeding companies Böhm-Nordkartoffel-Agrarproduktion GmbH & Co OHG (BNA), NORIKA Nordring-Kartoffelzucht- u. Vermehrungs-GmbH and SaKa Pflanzenzucht GmbH & Co. KG for seeds of cultivar crosses and seed tuber propagation. The donation of the seeds was part of the collaboration funded by GFPi.

## Conflict of Interest

The authors declare that the research was conducted in the absence of any commercial or financial relationships that could be construed as a potential conflict of interest.

## Supplementary Material

Data Sheet 1 Presentation 1 - Workflow of the selection experiment; Sup Figures and Sup Table 1 and 2.docx; Data 1 - Corrected normalized metabolome data of trials B2 and P3; Sup Table 3 - Forward and reverse primers for qRT-PCR; Data 2 - List of the expression values of 43 marker genes investigated in the samples used in this study.

## Abbreviations

2^−ΔCt^, Gene expression relative to the house-keeping genes; A_t_, Tolerant parent A; B2, B4, B6, Big-bag trials 2, 4 and 6; DRYM, Drought tolerance index deviation of the relative yield from the population median; DRYMp, Drought tolerance index deviation of the relative yield from the parent median; E_s_, Sensitive parent E; F1 - F8, Field trials 1 to 8; G1, Population of 600 potato lines before selection for above average yield; G2, Population of 200 potato lines selected from G1 for above average yield; GCMS, Gas chromatography mass spectrometry; LASSO, Least absolute shrinkage and selection operator; MAS; Marker assisted selection; MP_s_, Sensitive subpopulation selected based on tolerance predicted by model; MP_t_, Tolerant subpopulation selected based on tolerance predicted by model; P3, P5, P7, Pot trials 3, 5, and 7; PP_t_, Tolerant subpopulation selected based on yield from three stress trials; qRT-PCR, Quantitative reverse-transcription polymerase chain reaction; QTL, Quantitative trait loci; RH, Relative humidity; R_s_, Sensitive parent R; SI, Stress index; SSI, Stress susceptibility index; STI, Stress tolerance index; SY, Starch yield; T, Temperature; VPD, Vapour pressure deficit of the air.
