# Supplementary material for: Can Metabolite- and Transcript-Based Selection for Drought Tolerance in Solanum tuberosum Replace Selection on Yield in Arid Environments?
Source: Front Plant Sci. 2020 Jul 21;11:1071. doi: 10.3389/fpls.2020.01071 (PMC7385397; doi:10.3389/fpls.2020.01071)
Supplement: Data Sheet 1 — Presentation 1 - Workflow of the selection experiment; Sup Figures and Sup Table 1 and 2.docx; Data 1 - Corrected normalized metabolome data of trials B2 and P3; Sup Table 3 - Forward and reverse primers for qRT-PCR; Data 2 - List of the expression values of 43 marker genes investigated in the samples used in this study. [file DataSheet_1.zip › New folder/Sup Table S1_2_ Sup Figure S1_4.docx]

**Table S1**. **Pedigree for lines selected in subpopulations.** List of short name, selection rank within subpopulations PP_t_, MP_t_ and MP_s_, reference line id in the MPI-MP’s laboratory information management system (Köhl *et al.*, 2008)(GK_Sample_id), seedling name. Pedigree information: lines with a short name starting with EA originated from the seeds derived from a the cross Euroresa E_s_ x Albatros A_t_. lines with the short name starting with AR originate from the cross Albatros A_t_ x Ramses R_s_. The highlight colours in column C indicates the colour used in Figure 6 and Figure S4.

| C | Short name | PP_t_ | MP_t_ | MP_s_ | GK_Sample_id | seedling_name |
| --- | --- | --- | --- | --- | --- | --- |
|  | EA2 |  |  | 3 | 899440 | {[St.Euroresa.n].47-[St.Albatros.n].47}.2 |
|  | EA7 | 22 |  |  | 899445 | {[St.Euroresa.n].47-[St.Albatros.n].47}.7 |
|  | EA8 | 3 |  | 11 | 899446 | {[St.Euroresa.n].47-[St.Albatros.n].47}.8 |
|  | EA19 |  |  | 20 | 899457 | {[St.Euroresa.n].47-[St.Albatros.n].47}.19 |
|  | EA22 |  |  | 18 | 899460 | {[St.Euroresa.n].47-[St.Albatros.n].47}.22 |
|  | AR1 |  | 17 |  | 899464 | {[St.Albatros.n].25-[St.Ramses.n].9}.1 |
|  | AR21 | 24 |  |  | 899484 | {[St.Albatros.n].25-[St.Ramses.n].9}.21 |
|  | AR23 | 4 | 10 |  | 899486 | {[St.Albatros.n].25-[St.Ramses.n].9}.23 |
|  | AR28 | 19 |  |  | 899491 | {[St.Albatros.n].25-[St.Ramses.n].9}.28 |
|  | AR55 |  |  | 12 | 899518 | {[St.Albatros.n].25-[St.Ramses.n].9}.55 |
|  | AR56 |  | 22 |  | 899519 | {[St.Albatros.n].25-[St.Ramses.n].9}.56 |
|  | AR59 | 11 |  |  | 899522 | {[St.Albatros.n].25-[St.Ramses.n].9}.59 |
|  | AR67 |  | 20 |  | 899530 | {[St.Albatros.n].25-[St.Ramses.n].9}.67 |
|  | AR106 |  | 18 |  | 899569 | {[St.Albatros.n].25-[St.Ramses.n].9}.106 |
|  | AR121 | 8 | 3 |  | 899584 | {[St.Albatros.n].25-[St.Ramses.n].9}.121 |
|  | AR133 | 10 |  |  | 899596 | {[St.Albatros.n].25-[St.Ramses.n].9}.133 |
|  | AR157 |  | 2 |  | 899620 | {[St.Albatros.n].25-[St.Ramses.n].9}.157 |
|  | AR163 |  | 15 |  | 899626 | {[St.Albatros.n].25-[St.Ramses.n].9}.163 |
|  | AR183 |  | 14 |  | 899646 | {[St.Albatros.n].25-[St.Ramses.n].9}.183 |
|  | AR185 | 16 | 7 |  | 899648 | {[St.Albatros.n].25-[St.Ramses.n].9}.185 |
|  | AR196 | 6 | 12 |  | 899659 | {[St.Albatros.n].25-[St.Ramses.n].9}.196 |
|  | AR197 |  | 4 |  | 899660 | {[St.Albatros.n].25-[St.Ramses.n].9}.197 |
|  | AR200 |  | 13 |  | 899663 | {[St.Albatros.n].25-[St.Ramses.n].9}.200 |
|  | AR201 | 5 |  |  | 899664 | {[St.Albatros.n].25-[St.Ramses.n].9}.201 |
|  | AR202 | 9 |  |  | 899665 | {[St.Albatros.n].25-[St.Ramses.n].9}.202 |
|  | AR241 |  | 16 |  | 899704 | {[St.Albatros.n].25-[St.Ramses.n].9}.241 |
|  | AR245 |  | 9 |  | 899708 | {[St.Albatros.n].25-[St.Ramses.n].9}.245 |
|  | AR247 | 20 |  |  | 899710 | {[St.Albatros.n].25-[St.Ramses.n].9}.247 |
|  | AR254 |  | 19 |  | 899717 | {[St.Albatros.n].25-[St.Ramses.n].9}.254 |
|  | AR256 | 12 |  |  | 899719 | {[St.Albatros.n].25-[St.Ramses.n].9}.256 |
|  | AR269 |  | 1 |  | 899732 | {[St.Albatros.n].25-[St.Ramses.n].9}.269 |
|  | AR282 |  | 6 |  | 899745 | {[St.Albatros.n].25-[St.Ramses.n].9}.282 |
|  | AR285 | 18 | 11 |  | 899748 | {[St.Albatros.n].25-[St.Ramses.n].9}.285 |
|  | AR293 | 17 | 8 |  | 899756 | {[St.Albatros.n].25-[St.Ramses.n].9}.293 |
|  | EA28 |  | 23 |  | 899788 | {[St.Euroresa.n].47-[St.Albatros.n].47}.28 |
|  | EA54 |  |  | 10 | 899814 | {[St.Euroresa.n].47-[St.Albatros.n].47}.54 |
|  | EA55 | 21 |  | 4 | 899815 | {[St.Euroresa.n].47-[St.Albatros.n].47}.55 |
|  | Short name | PP_t_ | MP_t_ | MP_s_ | GK_Sample_id | seedling_name |
|  | EA62 | 15 |  |  | 899822 | {[St.Euroresa.n].47-[St.Albatros.n].47}.62 |
|  | EA71 |  |  | 22 | 899831 | {[St.Euroresa.n].47-[St.Albatros.n].47}.71 |
|  | EA74 |  | 5 |  | 899834 | {[St.Euroresa.n].47-[St.Albatros.n].47}.74 |
|  | EA87 |  | 21 |  | 899847 | {[St.Euroresa.n].47-[St.Albatros.n].47}.87 |
|  | EA92 |  |  | 5 | 899852 | {[St.Euroresa.n].47-[St.Albatros.n].47}.92 |
|  | EA111 |  |  | 13 | 899871 | {[St.Euroresa.n].47-[St.Albatros.n].47}.111 |
|  | EA112 |  |  | 2 | 899872 | {[St.Euroresa.n].47-[St.Albatros.n].47}.112 |
|  | EA131 | 7 |  | 19 | 899891 | {[St.Euroresa.n].47-[St.Albatros.n].47}.131 |
|  | EA45 | 23 |  |  | 899905 | {[St.Euroresa.n].47-[St.Albatros.n].47}.145 |
|  | EA154 |  |  | 14 | 899914 | {[St.Euroresa.n].47-[St.Albatros.n].47}.154 |
|  | EA162 | 2 |  |  | 899922 | {[St.Euroresa.n].47-[St.Albatros.n].47}.162 |
|  | EA165 |  |  | 6 | 899925 | {[St.Euroresa.n].47-[St.Albatros.n].47}.165 |
|  | EA172 |  |  | 9 | 899932 | {[St.Euroresa.n].47-[St.Albatros.n].47}.172 |
|  | EA173 |  |  | 8 | 899933 | {[St.Euroresa.n].47-[St.Albatros.n].47}.173 |
|  | EA174 |  |  | 15 | 899934 | {[St.Euroresa.n].47-[St.Albatros.n].47}.174 |
|  | EA200 | 14 |  |  | 899960 | {[St.Euroresa.n].47-[St.Albatros.n].47}.200 |
|  | EA208 | 13 |  |  | 899968 | {[St.Euroresa.n].47-[St.Albatros.n].47}.208 |
|  | EA252 |  |  | 7 | 900012 | {[St.Euroresa.n].47-[St.Albatros.n].47}.252 |
|  | EA264 | 1 |  |  | 900024 | {[St.Euroresa.n].47-[St.Albatros.n].47}.264 |
|  | EA269 |  |  | 1 | 900029 | {[St.Euroresa.n].47-[St.Albatros.n].47}.269 |
|  | EA273 |  |  | 21 | 900033 | {[St.Euroresa.n].47-[St.Albatros.n].47}.273 |
|  | EA279 |  |  | 16 | 900039 | {[St.Euroresa.n].47-[St.Albatros.n].47}.279 |
|  | EA280 |  |  | 17 | 900040 | {[St.Euroresa.n].47-[St.Albatros.n].47}.280 |

**Köhl KI, Basler G, Luedemann A, Selbig J, Walther D**. 2008. A plant resource and experiment management system based on the Golm Plant Database as a basic tool for omics research. Plant Methods **4**, 11.

**Table S2.** **Descriptive statistics on drought tolerance** DRYMp of the cultivars A, E, R, the population G2* (G2 minus the lines selected in the subpopulations) and the subpopulations PP_t_, MP_t_ and MP_s_ for the four test environments pot (3 experiments), big-bag (3 experiments) and field (7 experiments). M = mean, MD = median, SD = standard deviation.

| **Trialtype** | **G** | **M(DRYMp)** | **MD(DRYMp)** | **SD(DRYMp)** |
| --- | --- | --- | --- | --- |
| pot | A | -0.011 | -0.017 | 0.015 |
| pot | E | -0.019 | -0.016 | 0.015 |
| pot | R | 0.050 | 0.062 | 0.050 |
| pot | G2* | -0.035 | -0.037 | 0.075 |
| pot | PP_t_ | 0.007 | 0.005 | 0.050 |
| pot | MP_t_ | -0.003 | -0.008 | 0.055 |
| pot | MP_s_ | -0.016 | -0.014 | 0.056 |
| big-bag | A | 0.044 | 0.041 | 0.070 |
| big-bag | E | -0.015 | -0.031 | 0.037 |
| big-bag | R | -0.056 | -0.086 | 0.055 |
| big-bag | G2* | 0.083 | 0.054 | 0.239 |
| big-bag | PP_t_ | 0.093 | 0.074 | 0.180 |
| big-bag | MP_t_ | 0.030 | 0.014 | 0.119 |
| big-bag | MP_s_ | 0.015 | -0.005 | 0.148 |
| field | A | 0.043 | 0.053 | 0.047 |
| field | E | 0.000 | -0.005 | 0.066 |
| field | R | -0.005 | -0.030 | 0.056 |
| field | G2* | 0.049 | 0.057 | 0.098 |
| field | PP_t_ | 0.036 | 0.019 | 0.103 |
| field | MP_t_ | 0.019 | -0.004 | 0.098 |
| field | MP_s_ | 0.018 | 0.008 | 0.101 |

**Supplemental Figures**


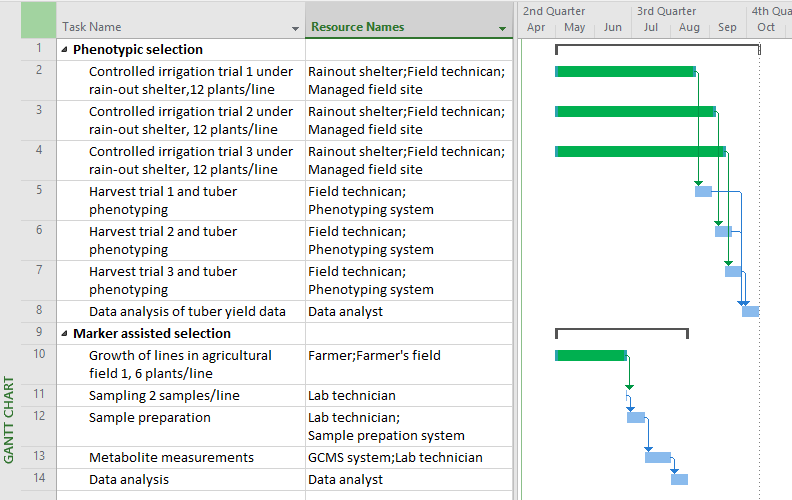


**Figure S1.** **GANT-Chart for selection procedures.** The duration of tasks shown in green is determined by environmental conditions. The duration of tasks shown in blue depends on amount of resources. Phenotypic selection requires at least three experiments with two treatment conditions. Samples for marker-assisted selection can be taken from plants grown under field conditions. Metabolite measurements can be replaced by transcript measurements by qRT-PCR.


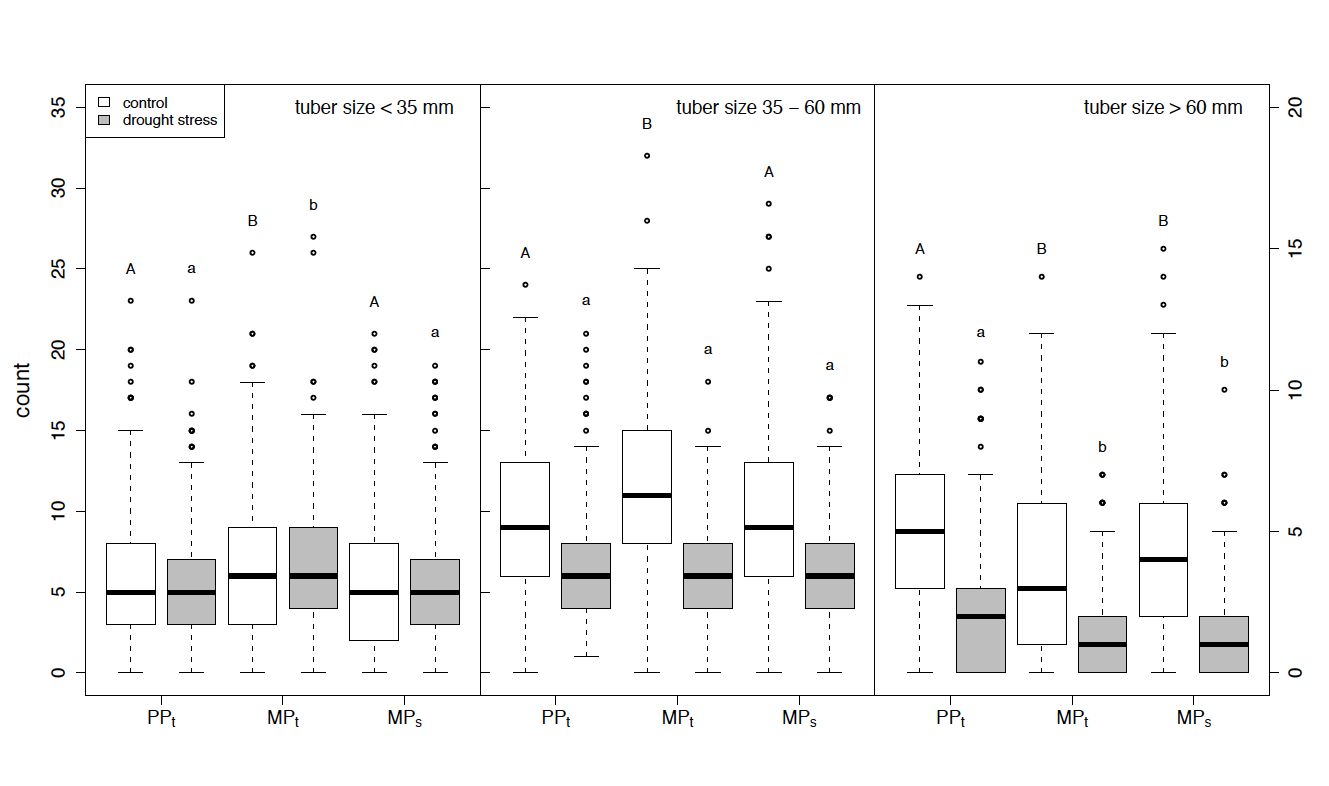


**FigureS2.** **Influence of treatment and subpopulation on the size distribution of potato tubers.** Distribution of the number of small (diameter < 35 mm), medium (35 to 60 mm) and big (> 60 mm) tubers in lines of subpopulations grown under optimal water supply (control) and drought stress. Data from big-bag trials B2, B4 and B6.


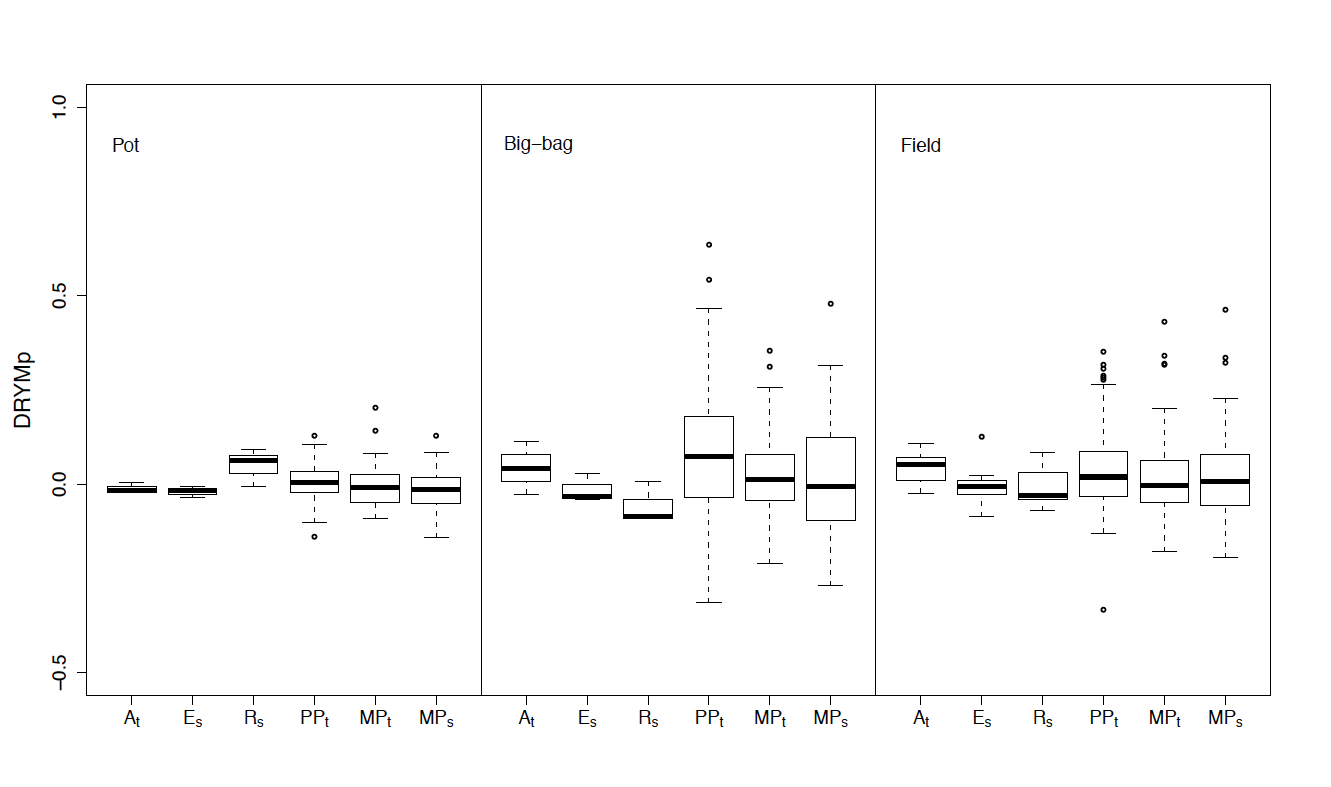


**Figure S3. Drought tolerance (DRYMp) in the three parent cultivars A_t_, E_s_ and R_s_ and the three subpopulations.** Distribution of average drought tolerance DRYMp in three subpopulations in pot trials (A), big-bag trials (B) and field trials (c) with significant treatment effect. The drought tolerance was normalized to the median of the relative starch yield of the three parents. Result of statistical analysis see table 3.


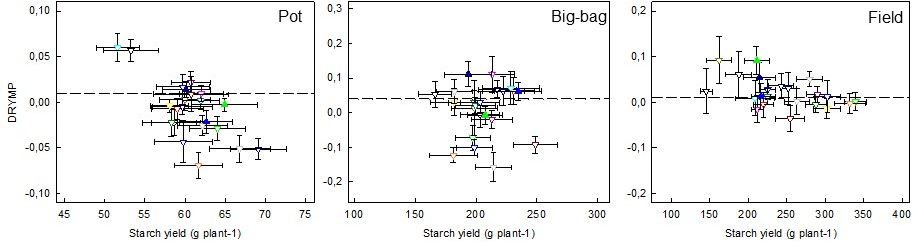


**Figure S4. Relationship between drought tolerance (DRYMp) and starch yield under optimal water supply in lines selected for inferior drought tolerance.** Mean and standard error (SE) of DRYMp depicted against mean and SE of tuber starch yield of the lines in MP_s_ in pot, big-bag and field trials. Tuber starch yield was normalized to account for the spatial effects in the experiments. The reference lines indicate the median of DRYMp in the respective test system. Those lines that are represented lines in PP_t_  and MP_s_ are represented by closed triangles and with the same colours used in Figure
