# Supplementary figures and images for: Can Metabolite- and Transcript-Based Selection for Drought Tolerance in Solanum tuberosum Replace Selection on Yield in Arid Environments?
Source: Front Plant Sci. 2020 Jul 21;11:1071. doi: 10.3389/fpls.2020.01071 (PMC7385397; doi:10.3389/fpls.2020.01071)

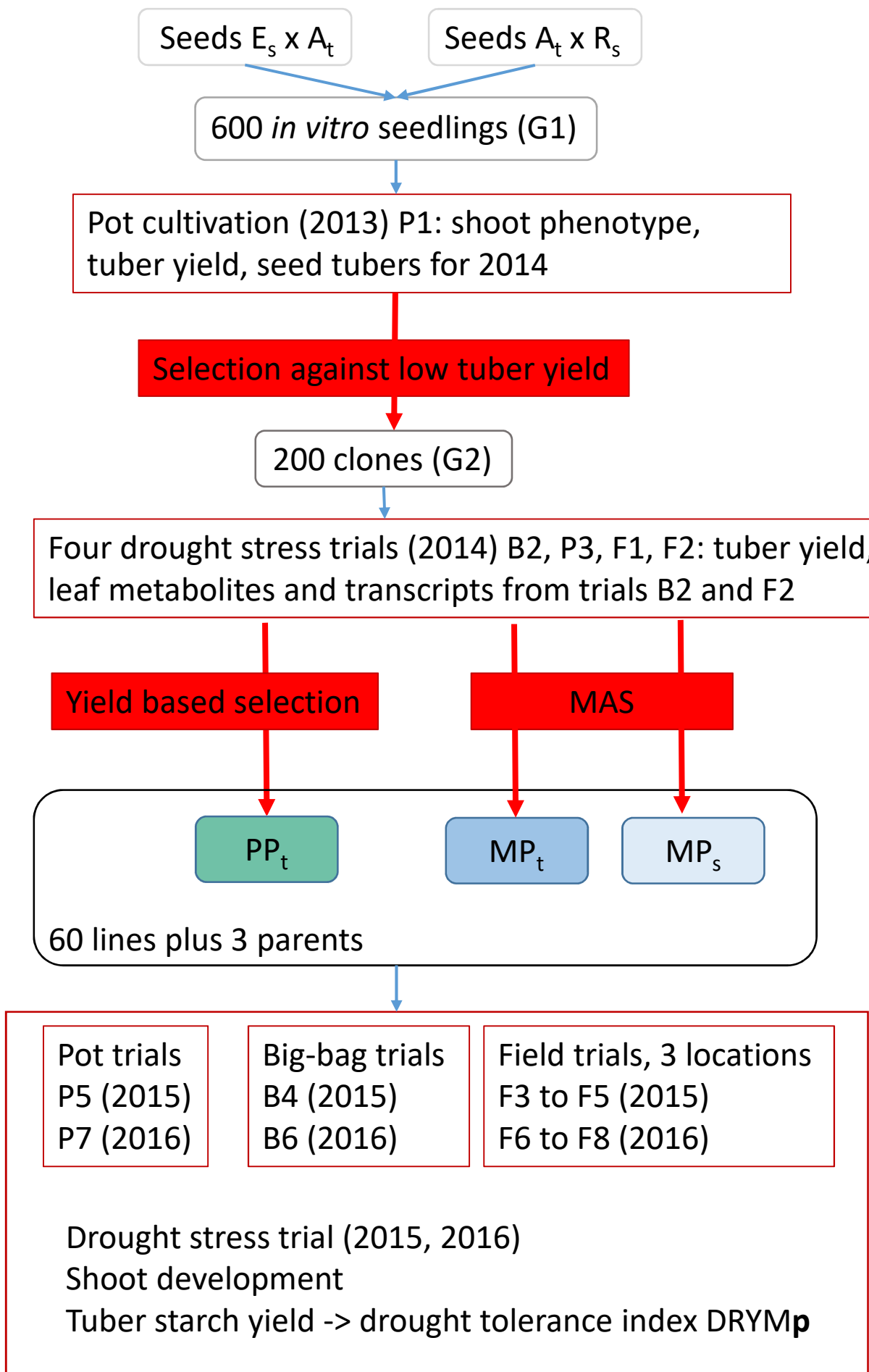

Supplement: Data Sheet 1 — Presentation 1 - Workflow of the selection experiment; Sup Figures and Sup Table 1 and 2.docx; Data 1 - Corrected normalized metabolome data of trials B2 and P3; Sup Table 3 - Forward and reverse primers for qRT-PCR; Data 2 - List of the expression values of 43 marker genes investigated in the samples used in this study. [file DataSheet_1.zip › New folder/Presentation 1.PDF]
